# Learning safe behaviour via justified human preferences and hypothetical queries

**DOI:** 10.1007/s10458-026-09770-8

**Published:** 2026-10-29

**Authors:** Ilias Kazantzidis, Timothy J. Norman, Yali Du, Christopher T. Freeman

**Affiliations:** 1 School of Electronics and Computer Science, University of Southampton, Building 32, University Road, Southampton, SO17 1BJ United Kingdom; 2 Department of Informatics, King’s College London, Bush House, 30 Aldwych, London, WC2R 2LS United Kingdom

**Keywords:** Safe Learning from Human Preferences, Safe Reinforcement Learning, Human-Agent Interaction, Human-Robot Interaction

## Abstract

Although reinforcement learning is a powerful paradigm for agent sequential decision-making, it cannot be used in its traditional form in most safety-critical environments. Human feedback can enable an agent to learn a good policy while avoiding unsafe states, but at the cost of human time. We present JPAL-HA, a model for safe learning in safety-critical environments that is grounded on two novel ideas: (i) human preferences over a choice of actions are augmented with *justifications* such as one action is preferred because the other is unsafe; and (ii) we use these justifications to guide the generation of future queries over *hypothetical actions* to enable the agent to more effectively map out unsafe regions. We evaluate JPAL-HA using modified configurations of the Island Navigation environment from the AI Safety Gridworlds. We empirically show that it significantly improves safety compared to existing approaches for safe learning from human preferences and outperforms other safe human-in-the-loop algorithms in terms of sample efficiency. We also include an analysis of safety/human burden trade-off. Additionally, we evaluate our method with people training a mobile robot, demonstrating that a diverse group of people can complete training in only a few minutes and that *hypothetical actions* not only further improve safety, but also slightly decrease the training time and keep it to admissible levels.

## Introduction

Inexperienced interaction with the real world has its dangers. Fortunately, in virtual environments an agent can simply ‘restart’ and no harm will be caused. This is the reason why traditional reinforcement learning (RL) has mastered some of the most difficult board [1] and video games [2, 3], where we do not care about continuous blundering of queens in chess or deaths of virtual creatures. In real-world applications, such as autonomous vehicles and robotics, however, we do mind if a self-driving car (agent) crashes even once into an obstacle or even worse a pedestrian (environment). Such examples motivate the development of safe RL, where in addition to the pursuit of the optimal policy, safety constraints have to be respected [4]. The main problem safe RL tries to address is that the agent cannot naively take exploratory actions in order to gain new information. This *safe exploration* problem, combined with the problem of catastrophic forgetting, where the agent gradually forgets things it previously learnt, makes safe RL a very difficult task with, at present, no general solution.

Numerous techniques have been proposed to address this problem, such as those augmenting the objective function with constraints in the single-agent domain [4–9] or those that incorporate norms in the multi-agent domain [10]. Our solution lies in the context of techniques that involve a human in the learning process who teaches the agent through human preferences. Our proposed algorithm, called **J**ustified Human **P**references for **A**ctive **L**earning with **H**ypothetical **A**ctions (**JPAL-HA**), builds on the *Parenting* approach [11] which also uses the technique of human preferences (the human providing feedback to the agent by expressing a preference between two state–action pairs) and learns directly in a supervised way from the preferences. We augment human preferences with two novel ideas:

1. *Justifications*: a binary warning signal expressed by a human alongside a preference between two actions, such as one action is preferred because the other is unsafe. Justifications are simple ‘Yes’ or ‘No’ answers and may require marginally more time for a human than asking for a preference. However, their impact on safety is significant.
2. *Hypothetical Actions* (*HAs*): we use justifications to guide the generation of future queries regarding hypothetical actions that could have been taken, in order to enable the agent to more effectively map out unsafe regions.

As discussed in previous research [12, 13], in JPAL-HA, in contrast to traditional RL, the ‘reinforcement’ is not the traditional environmental reward. First, in many real-world tasks, such as robotics, the goals are complicated and such rewards are hard or even impossible to design. Moreover, even if they exist, they may be sparse or delayed. Additionally, traditional RL is, in general, very sample-inefficient (sometimes needing millions of steps), which makes training impossible in the real world where every step may not take just milliseconds as in simulations. Further, even if there is a well-defined reward function to be optimised, the final agent behaviour will not always align with what the human really wants the agent to do (constituting an *agent alignment* or *reward specification* problem [14]). Finally, and most importantly, due to safe exploration, environmental rewards may also not be helpful in real-world safety-critical domains such as robotics; i.e. informing an agent-robot that ‘you got -100 points for destroying your gripper, the assembly and harming a person’, is obviously not desirable.

In JPAL-HA, the reinforcement comes from human feedback before an action is taken, and is not in the form of a numerical reward value; instead it takes the form of preference and justification answers. Moreover, we do not use these preferences to build a reward model but to learn directly from an increasing dataset of human preferences with supervised learning. These choices are discussed in detail in Section 6.1 with comparison to related work.

Although obtaining human guidance improves the performance and safety of the algorithm, it introduces the issue of human burden. Overall, we assess the general efficiency of an agent learning approach by three factors: (i) performance, i.e. does the algorithm find the optimal or a near-optimal policy; (ii) sample (or data) efficiency, i.e. how much time training takes; and (iii) safety, i.e. how many unsafe events occur during training before the agent has learnt the optimal policy. Performance is evaluated by the return, and safety by the number of catastrophic actions. Sample efficiency would normally be measured by the number of overall interactions (i.e. steps) until the optimal policy is found. However, when a human is employed, what takes additional time is the response time of the human. Hence, for sample efficiency we also consider the human burden; i.e. how many questions are asked to the human (and how hard they are) until optimality is achieved.

The contributions of this work are:

- We present in detail two new ideas that can be generalised and incorporated in models that use human preferences. The first is learning with *justifications*, which does not depend on the general framework of JPAL-HA or a specific environment. Its benefit is reflected by significantly improving safety. The second is *HAs*, which follows justifications and further improves safety. It also significantly compensates for human burden, assuming that justifications and HAs place a marginal additional cost.
- We introduce a novel algorithm called JPAL-HA, a learning-from-human-preferences approach, which incorporates both of the aforementioned features of justifications and HAs. We empirically show that JPAL-HA significantly improves safety during training relative to existing approaches for safe learning from human preferences, and achieves better sample efficiency than other safe human-in-the-loop algorithms. Also, the user can tune the level of safety they require based on a hyperparameter that trades safety with human burden.
- We evaluate our algorithm not only in simulation environments but also in a real-world mobile-robot task, achieving successful learning in only a few minutes by a diverse group of people. We also demonstrate comparisons made between different methods in this real-world task.

The *justifications* idea is introduced on the *JPAL* algorithm (Section 4.1), which builds on the Parenting algorithm [11] (Section 3); the technique of *hypothetical actions* is introduced on *JPAL-HA* (Section 4.2), which builds on JPAL. Before presenting these models, we provide an overview of the safe exploration problem and how we model it in this paper.

## The safe exploration problem

Safe exploration [4] is the key challenge for safe RL. One of the fundamental concepts of RL is balancing exploitation with exploration during learning. Unrestricted exploration can, however, lead to unsafe states, or even catastrophic failures in the environment due to the agent’s action.

**Fig. 1:**
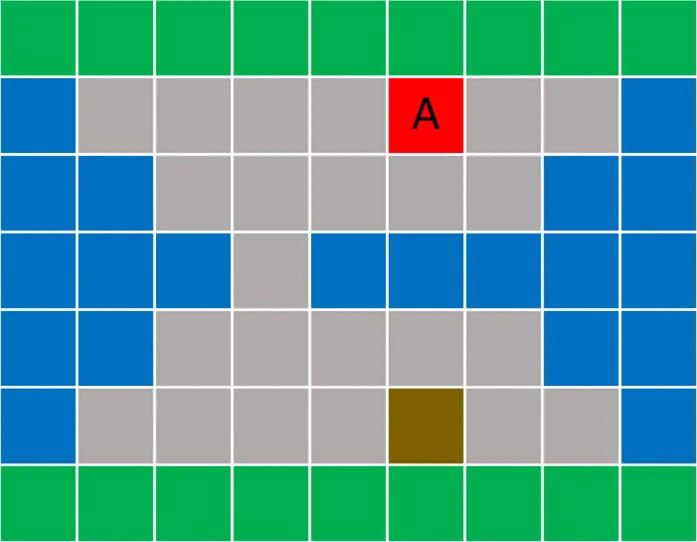
The Island Navigation environment. **A**: Agent, **Red**: Initialisation cell, **Gold**: Goal, **Blue**: Water, **Green**: Wall, **Grey**: Free road

Leike et al. [15] represent this problem with a gridworld environment, which, despite its small state space, captures the semantics of safety well. The environment, called Island Navigation, was adapted in [11] to the more challenging version illustrated in Fig. 1. We further created an arena based on this simulated environment for training a mobile robot using real human input, presented in Fig. 9. The agent *A* has four moves: UP, DOWN, LEFT and RIGHT, is initialised in the red cell and tries to reach the goal cell (gold). For every step it takes, it receives a -1 reward from the environment, and if it reaches the goal state it receives a reward of +50. There are cells of free road (grey). If the agent ‘hits’ a wall cell (green) it does not move, but receives -1 reward. The agent cannot swim and ‘dies’ when entering a water cell (blue), and the episode ends with an added reward of -50. In this work, we do not formulate safety as a constrained Markov decision process (CMDP) constraint [16]. Instead, we define an unsafe event as the execution of an action that transitions the agent into an unsafe state (water cell) and causes harm (a death). Thus, in our paradigm, safe exploration is understood as minimising the number of unsafe events until the agent has learnt an optimal policy for the task. Our goal is to minimise such training violations until the agent is ready for deployment, rather than to prove that they are impossible in all domains; highly safety-critical domains may require near-zero tolerance, while less critical ones may allow a controlled amount of training risk.

We study the above problem in the following setting. First, the unsafe states (water) are unknown to the agent and we have access to neither the state transition dynamics (i.e. there is no available simulator to sample trajectories^1^), nor to the handcrafted reward function described above^2^. This represents a challenging real-world setting. The only source of information is the human trainer (user), who must have a reasonable understanding of the environment (can correctly answer all preference and justification queries — simple for the Island Navigation environment, as we will see later) and recognise unsafe states. With the latter assumption, a number of important safety-critical applications can be tackled. However, in very complex applications, such as helicopter flight, effective teaching would rely on the knowledge of a limited number of expert human trainers, who would be able to provide reliable input. Additionally, we assume that the agent can pause its task to query the user before taking action. While this is feasible in controlled environments, it may limit applicability in domains like autonomous driving, where uninterrupted operation is critical. In such cases, complementary resources like fallback safety mechanisms may be required alongside our method to ensure safe behaviour in real-time, continuous environments. Moreover, although the method proposed in this paper was designed for problems with discrete action spaces, extending its core ideas beyond that is discussed in Section 6.3. Finally, querying the user is expensive, for which we present a detailed safety-human burden trade-off analysis in Section 5.4.

Specifically, for the Island Navigation environment we also assume that the agent can sense (observe) its exact location (coordinates x, y) and the whole state of the board. In other settings, we could assume that the agent (i.e. a robot) uses an attached camera to obtain the observations [17]. Finally, here we do not consider any debatable (normative) cases, i.e. different opinions regarding the correct human input. This could occur in some cases and needs extra modelling [18]; however, it goes beyond the scope of this paper.

## The Parenting approach

Our newly-proposed methods, JPAL and JPAL-HA, build upon the parent-child (human-agent) intuition of the *Parenting* model [11]. The child (untrained agent) is unfamiliar with the dangers of most states and asks the parent (the human) a lot of questions. The more familiar the child becomes with a state, the fewer queries it needs to ask about it. As the child develops experience, it may take actions independently without asking the parent when it is confident about the safety of its action. The child asks for the parent’s preference between two actions that they think are the best amongst all possible actions. Table 1 lists the symbols of the Parenting, JPAL and JPAL-HA algorithms to be presented.

**Table 1:** Symbols used in Parenting, JPAL and JPAL-HA algorithms. In Parenting, all symbols from $s_t$ to$p_{{ATFQ}}$ (reading down the first column) are used. JPAL adds $J_P$ (from the second column), and JPAL-HA introduces the remaining symbols

Symbol	Description	Domain/Range	Symbol	Description	Domain/Range
$s_t$	Current agent’s state	-	$p_{{REC}}$	Probability of recording in $\mathcal {R}$	[0, 1]
$f(s_t)$	Number of queries from $s_t$	$\mathbb {N}$	ATFQ	After-The-Fact Query	-
$a_t^{(0)}, a_t^{(1)}$	Initially sampled actions	-	$p_{{ATFQ}}$	Probability of asking an ATFQ	[0, 1]
$\pi (a\!\mid \!s)$	Agent’s policy model	[0, 1]	$J_P$	Justification over preference *P*	{w, n}
BTFQ	Before-The-Fact Query	-	$\mathcal {S}$	Set of safe actions	-
$\mathcal {X}$	Embraced memory	-	$\mathcal {C}$	Set of catastrophic actions	-
$p_{{BTFQ}}$	Hyperparameter for BTFQ	[0, 1)	*A*	Action space size	$\mathbb {N}$
*P*	Human preference		$\alpha _{{safe}}$	Min. safe action space fraction	(0, 1]
	for Parenting	$\{{1^{\text {st}}}, {2}^{\text {nd}}, {either}, {neither}\}$	*k*	Safe-to-catastrophic actions ratio	[1, A-1]
	for JPAL/JPAL-HA	$\{{1}^{\text {st}}, {2}^{\text {nd}}, {equal}\}$	$\alpha _{{stop}}$	Explored actions upper fraction	(0, 1]
*μ *	Preference label		$a_{{best}}$	Best action in $\mathcal {S}$	-
	for Parenting	$\{0, 0.5, 1\}$	$a_{{new}}$	New (hypothetical) action	-
	for JPAL/JPAL-HA	$\{0, 0.25, 0.5, 0.75, 1\}$	$J_{a_{{new}}}$	Single justification over $a_{{new}}$	{w, n}
$a^*$	Final chosen action	-	$P_{{add}}$	Additional pref. on $\langle a_{{new}}$, $a_{{best}} \rangle$	$\{{1}^{\text {st}}, {2}^{\text {nd}},$* equal**\}*
$\mathcal {R}$	Temporary memory	-	DP	Deadly pattern	-

Algorithm 1 presents Parenting more formally^3^. The goal is to train a policy model $\pi (a\!\mid \!s) \in [0,1]$, i.e. the probability of choosing action *a* from state *s*, from which the agent then picks the greedy action and acts on the environment during deployment. The model is the average of: (i) a convolutional neural network (CNN) with input being the state of the board; and (ii) a feed-forward neural network (FNN) with input being the type of the four neighbouring cells (in Up, Down, Left and Right directions). It is trained by supervised learning from human preferences stored in an *embraced memory *$\mathcal {X}$. For our learning paradigm, the output has four neurons as the number of possible actions. The agent does not use any further help or input from the human during deployment.

In each step of the algorithm and until the optimal policy has been found (Lines 6–16), the agent, from state $s_{t}$, asks a *Before-The-Fact-Query (BTFQ)*, i.e. a query issued to the human before taking an action, with probability $(p_{{BTFQ}})^{f(s_{t})}$, where $f(s_{t})$ (*familiarity*) is the number of queries that have been issued from $s_{t}$ and $p_{{BTFQ}}\in [0,1)$ is a hyperparameter normally set close to 1, to ensure the agent is cautious in new states (Lines 7–8). Note that $\left( p_{{BTFQ}}\right) ^{f(s_{t})}$ gradually decreases as the agent asks more queries from $s_{t}$, reducing human burden. Although defining $p_{{BTFQ}}$ in [0, 1) and decreasing it by the number of queries issued from a specific state is a practical method for the evaluation in the Island Navigation gridworld, Appendix A presents a more general formula that can be applied to larger and continuous spaces.

BTFQs are constructed by the agent sampling two distinct actions $a_{t}^{(0)}$ (${1^{\text {st}}}$ sampled action) and $a_{t}^{(1)}$ (${2}^{\text {nd}}$ sampled action) from $\pi (a \!\mid \! s)$ and querying the human. The human answers with a preference $P\in \{{1}^{\text {st}}, {2}^{\text {nd}}, {either}, {neither}\}$, where ${1}^{\text {st}}$ and ${2}^{\text {nd}}$ means preference for the first or the second sampled action, respectively; *either* means that both actions are equivalently preferable and safe (not necessarily the best ones though); and *neither* means that both actions are catastrophic (Line 2). The agent from *P* can simply find the recommended action $a^{*}$ which will be executed (Lines 3, 5). If $P={either}$, a random action between the two is executed; if $P={neither}$, then resampling (without replacement in any order of the combination of actions $a_{t}^{(0)}$ and $a_{t}^{(1)}$) takes place from $\pi (a \!\mid \! s)$ until a non-catastrophic action is identified. Moreover, the human’s input *P* drives the agent’s learning. In order to do that, the preference ground-truth label *μ* is derived, with *μ* denoting how much the first action is preferred to the second, and *1-μ* denoting the opposite. Here, $\mu \in \{0, 0.5, 1\}$, where 1 and 0 represent the preference for the ${1^{\text {st}}}$ and ${2}^{\text {nd}}$ action, respectively, and 0.5 means no preference (Line 3). Then, the entry ($s_{t}$, $a_{t}^{(0)}$, $a_{t}^{(1)}$, *μ*, $a^{*}$) is stored in $\mathcal {X}$, from which the agent will learn at the end of the step (Line 4)^4^.

If a BTFQ is not asked (meaning the agent ‘knows the state well’, and so can take a safe action with high probability) (Lines 9–14), then Parenting continues in the same manner as JPAL and JPAL-HA at this point. It potentially *records and asks an After-The-Fact-Query (ATFQ)* (Lines 10–13)^5^ and it executes the greedy action from $\pi (a\!\mid \! s)$ (Line 14). A step finishes by learning via supervised learning from the human preferences stored in $\mathcal {X}$ (Line 15). Before giving the details of Recording/Asking ATFQs and Learning (Section 4.3), we introduce the novel features of JPAL and JPAL-HA.

**Algorithm 1:**
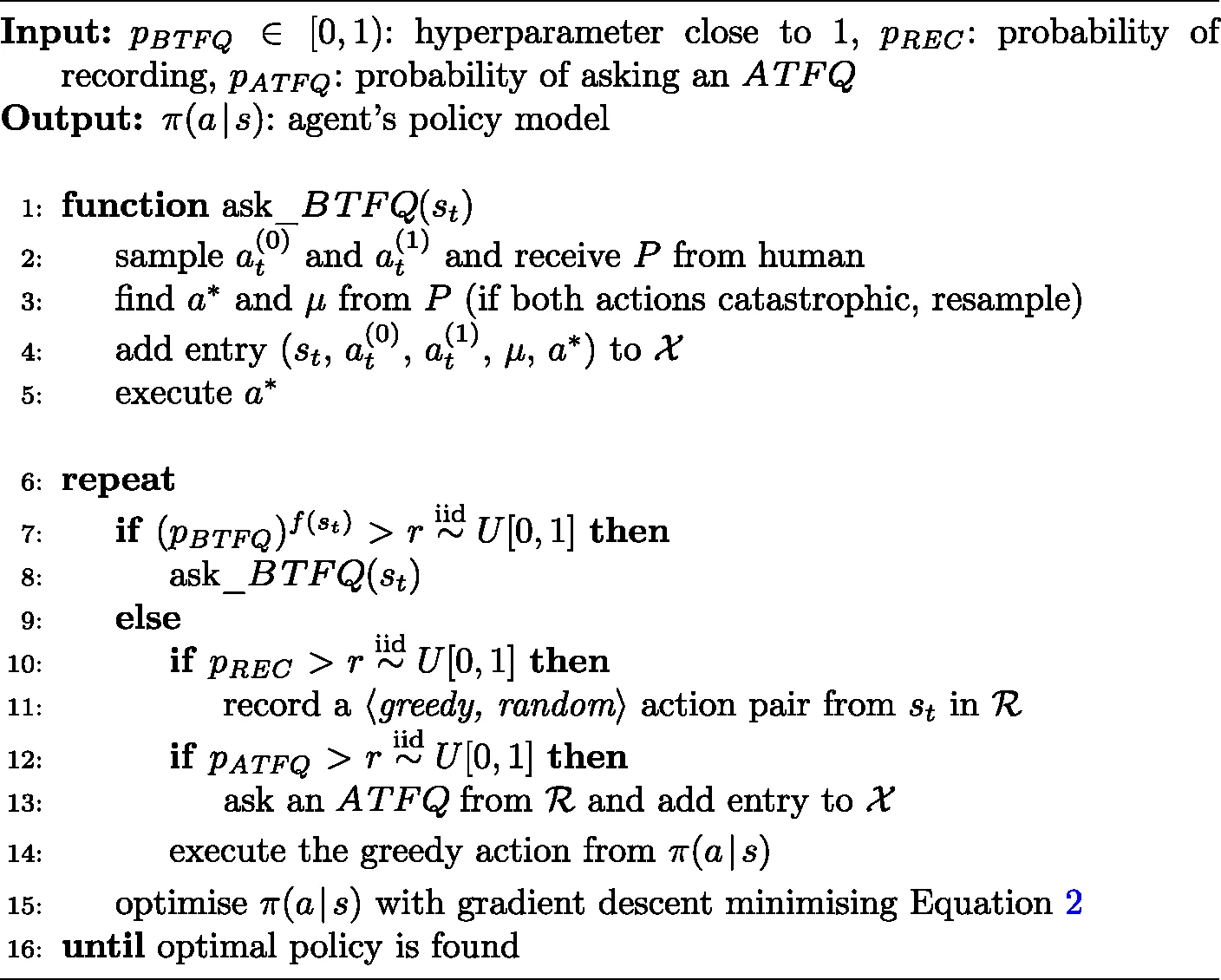
Parenting (optimised)

## Learning with justifications and hypothetical actions

The ideas that we add to the parent-child analogy of Section 3 are: (i) *justifications*; and (ii) *Hypothetical Actions*. Regarding (i), the parent not only gives advice about which action is better but also warnings (a kind of justification for the expressed preference^6^) regarding safety. That is, if one of the actions proposed is catastrophic, it is valuable for the child to know. Providing this guidance is usually a small additional effort for the parent, but helps to promote safer behaviour. Regarding (ii), if a justification is ‘warning’, which means that the child asked a preference query that included at least one catastrophic action, that predisposes the child to think that more actions could lead to a catastrophe from that state (‘where there’s smoke, there’s fire’). So the child may want to ask further questions regarding *hypothetical actions* that could have been taken from that state, and as such map out potentially unsafe regions of the environment faster. This is beneficial not only for the child’s safety, but also so that the parent’s effort can be more focused on dangerous regions. Moreover, the child, when faced with a state of multiple unsafe behaviours, creates and stores ‘deadly patterns’ and is always suspicious about them, in order both to avoid them in another state and not to bother the parent again. A deadly pattern could be, for example, the combination of moves (Left, Right) when crossing a bridge. Both actions would lead to the cliff and the child should be aware of it as soon as possible, both for its safety and for not continuously bothering the parent.

### JPAL

More formally, in JPAL (Algorithm 2), $P\in \{{1^{\text {st}}}, {2}^{\text {nd}}, {equal}\}$, where ${1^{\text {st}}}$ and ${2}^{\text {nd}}$ means preference for the first or the second sampled action, respectively; and *equal* means no preference (either because they are equivalently preferable and safe, or because they are both catastrophic). However, in addition to a plain preference *P* (as in Parenting), the agent requests and gets a justification over this preference $J_{P}\in \{w, n\}$, where *w* means ‘warning’, i.e. at least one proposed action is catastrophic (i.e. leads into water), and *n* means ‘no warning’, i.e. both actions are safe (Line 2).

The agent from *P* can firstly again find $$a^{*}$$ (Line 3). However, in JPAL (and JPAL-HA), in contrast to Parenting, we can make a more efficient mapping $$P \times J_{P} \rightarrow \mu$$, where $$\mu \in \{0, 0.25, 0.5, 0.75, 1\}$$ (Line 3), that directly improves the safety of the agent.^7^ We use *μ =1* to denote total preference for the $${1^{\text {st}}}$$ action implying a catastrophe for the $${2}^{\text {nd}}$$ one (i.e. $$P={1^{\text {st}}},J_{P}=w$$), *μ =0.75* to denote simple preference for the $${1^{\text {st}}}$$ action (i.e. $$P={1^{\text {st}}},J_P=n$$), *μ =0.5* to denote equal preference (i.e. $$P={equal}, J_{P}:arbitrary)$$ and *μ =0* and 0.25 similarly to indicate preference for the $${2}^{\text {nd}}$$ action. In the case that the actions are equivalent and safe (i.e. $$P={equal}$$, $$J_{P}=n$$), a random action between the two is executed, while if they are both catastrophic (i.e. $$P={equal}$$, $$J_{P}=w$$), we resample until a non-catastrophic action is identified.^8^ Having mapped *P* and $$J_{P}$$ to *μ*, we store in $$\mathcal {X}$$ the entry: ($$s_{t}$$, $$a_{t}^{(0)}$$, $$a_{t}^{(1)}$$, *μ*, $$a^{*}$$) (Line 4). Finally, $$a^{*}$$ is executed. At this point, JPAL continues the same as Parenting and JPAL-HA (Section 4.3).

**Algorithm 2:**
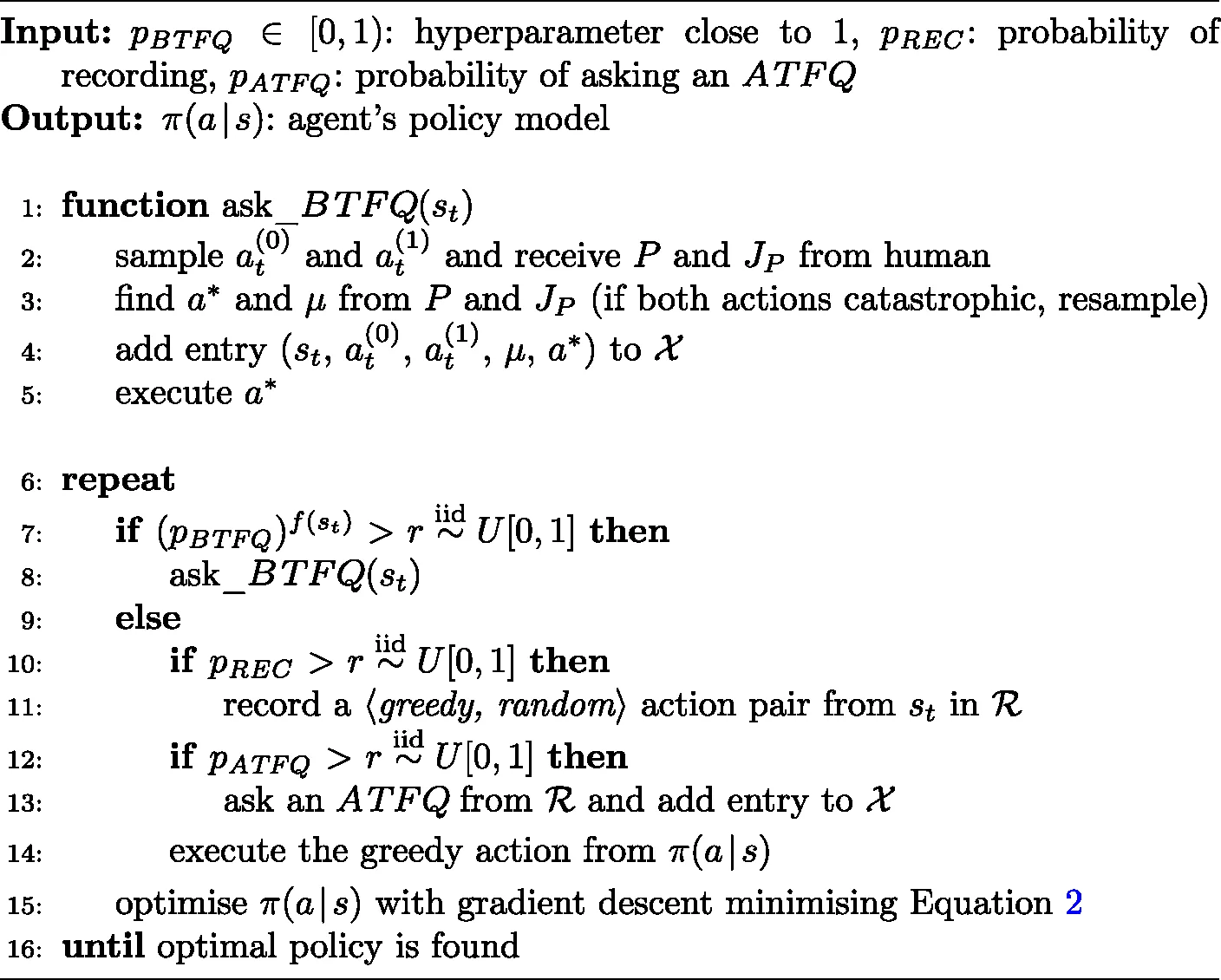
JPAL

### JPAL-HA

**Fig. 2:**
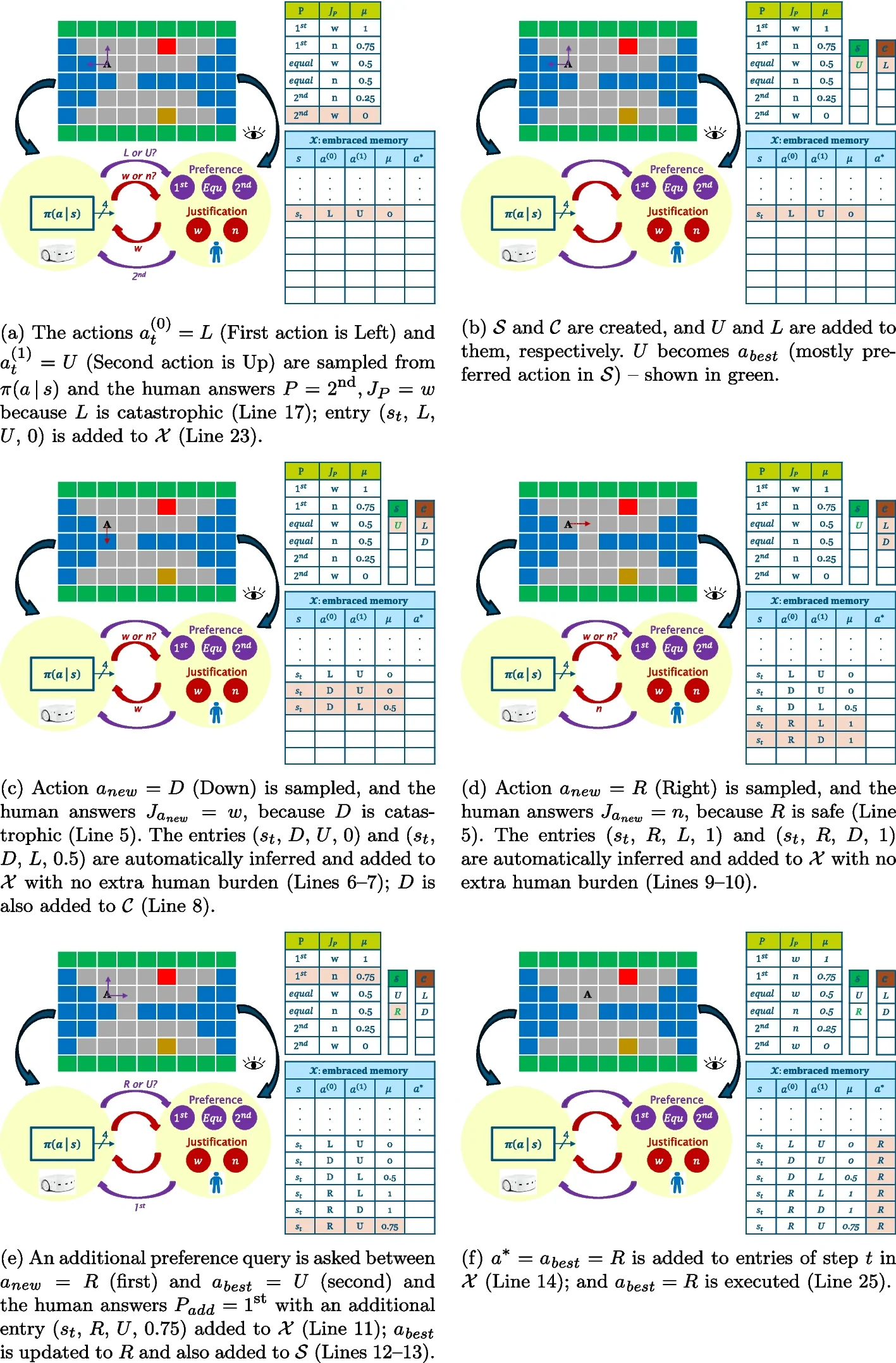
Example of JPAL-HA (Algorithm 3) for a specific state $s_{t}$. The mapping $P \times J_{P} \rightarrow \mu$, and the interactions amongst the agent, the human and the environment are depicted, along with the evolution (from Fig. 2a to f) of the embraced memory $\mathcal {X}$, the set of safe actions $\mathcal {S}$ and the set of catastrophic actions $\mathcal {C}$

**Fig. 3:**
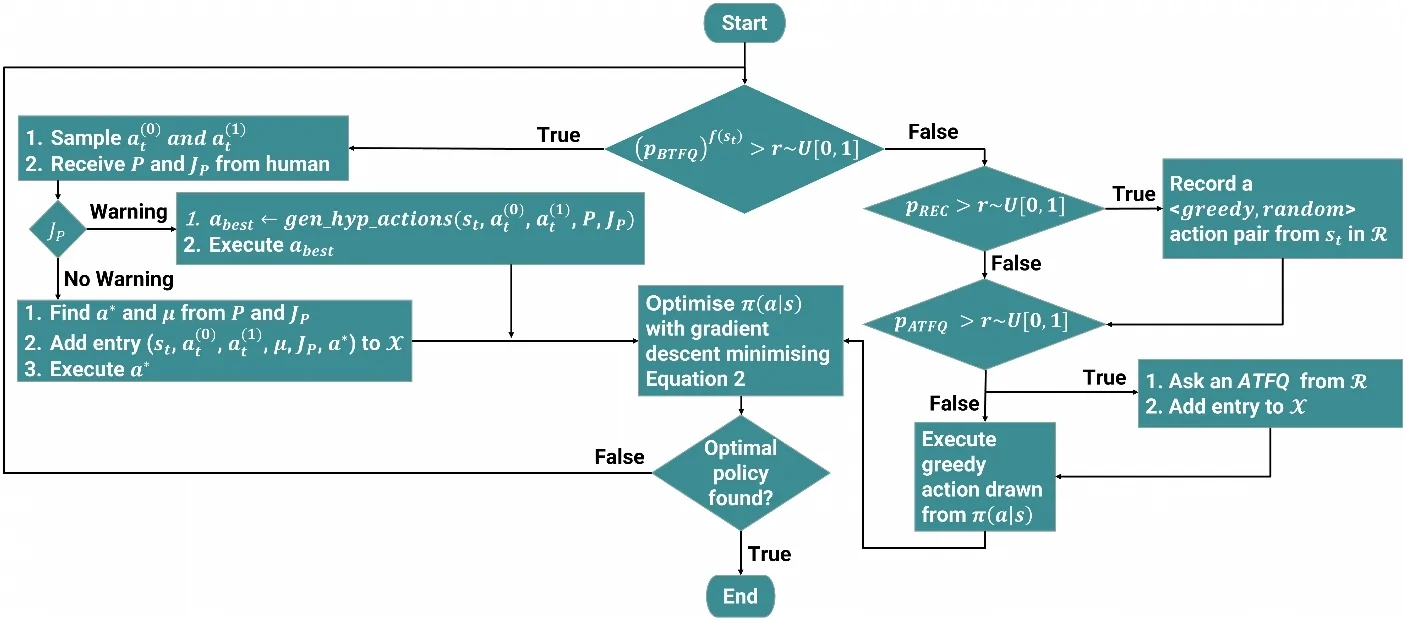
JPAL-HA flowchart

As previously stated, JPAL-HA (Algorithm 3) introduces the idea of hypothetical actions and builds on JPAL. Figure 2 shows a characteristic example involving the idea of hypothetical actions and Fig. 3 illustrates the flowchart of the complete JPAL-HA algorithm. The description in Section 4.1 holds for JPAL-HA, except for the case of $J_{P}=w$. Specifically, if $J_{P}=n$, then JPAL-HA works exactly like JPAL (Lines 18–21). However, if $J_{P}=w$ (at least one of $a_{t}^{(0)}$ or $a_{t}^{(1)}$ is catastrophic), the agent will find *μ* from *P* and $J_{P}$ as in JPAL, but it will not do the same for $a^{*}$. Instead, it will add only ($s_{t}$, $a_{t}^{(0)}$, $a_{t}^{(1)}$, *μ*) to $\mathcal {X}$ (leaving $a^{*}$ blank temporarily — we will see why), and then *generate hypothetical actions* (Lines 22-24). Specifically, it creates a set of safe actions $\mathcal {S}$ and a set of catastrophic actions $\mathcal {C}$, where initially $a_{t}^{(0)}$ and $a_{t}^{(1)}$ are added based on whether they are safe or not; this can be determined automatically by combining *P* and $J_{P}$ (Lines 2–3). Then the following loop (Lines 4–13) is executed: a new action $a_{{new}}$ (*hypothetical action*) is sampled and *only* a single justification over the new action $J_{a_{{new}}}\in \{w, n\}$ is asked from the human, where *w* means ‘warning’ ($a_{{new}}$ is a catastrophe) and *n* means ‘no warning’ ($a_{{new}}$ is safe) (Line 5). If $J_{a_{{new}}}=w$, then $\mathcal {X}$ is extended to include all the combinations of $a_{{new}}$ and the actions already in $\mathcal {S}$ and $\mathcal {C}$ — leaving $a^{*}$ blank temporarily^9^ (Line 7). Crucially, since we know that one action is already catastrophic, no extra human input is needed for these added entries to $\mathcal {X}$, i.e. *μ* and $J_{P}$ of the new entries can be inferred automatically. Additionally, $a_{{new}}$ is added to $\mathcal {C}$ (Line 8). If $J_{a_{{new}}}=n$, then $\mathcal {X}$ can again be automatically extended to include the combinations of $a_{{new}}$ and the actions already in $\mathcal {C}$ — leaving $a^{*}$ blank temporarily (Line 10). However, regarding $a_{{new}}$ and the actions already in $\mathcal {S}$, an additional preference query $P_{{add}}$ is asked between $a_{{new}}$ and the best (most preferred) action in $\mathcal {S}$ until that loop iteration $a_{{best}}$, leading to another entry in $\mathcal {X}$ — again leaving $a^{*}$ blank temporarily (Line 11). Potentially $a_{{best}}$ can now be substituted with $a_{{new}}$ in case $a_{{new}}$ is preferred (Line 12). Finally, $a_{{new}}$ is added to $\mathcal {S}$ since it is safe (Line 13).

We create a general formula to stop the loop (Lines 4–13) which considers initially that a minimum portion $\alpha _{{safe}} \! \in \!(0, 1]$ of the number of safe actions $\mid \!\! \mathcal {S} \!\! \mid$ stored in $\mathcal {S}$ relative to the number of all possible actions *A* should be discovered. Once it is discovered, then the loop stops when the safe-to-catastrophic actions ratio $k \! \in \! [1, A-1]$ is met. However, if this ratio is not reached after some iterations and we do not want to investigate every possible action, then the loop could stop when a portion $\alpha _{{stop}} \! \in \! (0, 1]$ of the number of all explored actions ($\mid \!\!\mathcal {S} \!\!\mid$ + $\mid \!\!\mathcal {C}\!\! \mid$) relative to *A* has been covered. Thus, this can be expressed as the stopping formula for the loop:

1 $$\begin{aligned} \frac{\mid \mathcal {S} \mid }{A} \ge \alpha _{{safe}} \ {{and}} \ \left( \frac{\mid \mathcal {S}\mid }{\mid \mathcal {C} \mid } \ge k \ {{or}} \ \frac{\mid \mathcal {S}\mid + \mid \mathcal {C} \mid }{A} \ge \alpha _{{stop}} \right) , \end{aligned}$$

or the equivalent for continuation shown in Line 4. $\alpha _{{safe}}$ ensures a minimum safety, *k* represents the confidence that the agent can stop since it has found enough safe actions relative to catastrophic, and $\alpha _{{stop}}$ ensures that in larger action spaces, the agent does not exhaustively search every action, but stops after exploring a representative upper limit. These hyperparameters can be tuned based on empirical results and knowledge across different environments.^10^ When the loop finishes, the correct action $a^{*}$ added to $\mathcal {X}$ is equal to $a_{{best}}$ for all the combinations of actions that were involved during the loop (Line 14) and $a_{{best}}$ is also executed (Line 25). Overall, the aim of HAs is to (i) more quickly investigate a dangerous area with minimal user input and without interrupting them repeatedly for the same state; and (ii) potentially improve the executed action, and thus find the goal state quicker.

Additionally, the procedure of HAs is optionally augmented with the mechanism of dynamically *detecting deadly patterns*. Specifically, when a combination of at least two catastrophic actions from the same state is detected (whether from the beginning of two catastrophic sampled actions or during the run of the loop), then a deadly pattern *dp* is created and stored in the *danger memory*$\mathcal {D}$. Memory $\mathcal {D}$ is usually small and contains patterns that will be checked both during training to avoid unnecessary queries to the human and during deployment as safety constraints. Essentially, $\mathcal {D}$ is a set of dynamically created constraints that have to be respected throughout training and deployment, such as ‘do not go left or right when crossing a bridge’.

### Recording/Asking ATFQs and learning

Occasionally, one may expect a child to ask slightly ‘unconventional’ random questions to a parent in order to explore new possibilities that may not have been investigated yet. This mechanism of *recording and asking ATFQs* was inspired by [11], but it was significantly modified and improved. It is also applied the same way for all Parenting, JPAL and JPAL-HA. Specifically, continuing the algorithms (Lines 9–15 in Parenting and JPAL, and 29–34 in JPAL-HA), if the agent does not ask a BTFQ, it will select the greedy action from $\pi (a\!\mid \! s)$. Before executing it (Line 14 in Parenting and JPAL, and 34 in JPAL-HA), updating its policy (Line 15 in Parenting and JPAL, and 35 in JPAL-HA) and finishing the step, it performs two more tasks. First, it records a pair of the greedy action and a randomly chosen one and adds it to a *temporary memory*$\mathcal {R}$ with probability $p_{{REC}}$ (Lines 10–11 in Parenting and JPAL, and 30–31 in JPAL-HA). Second, regardless of recording in that step, it could also, with probability $p_{{ATFQ}}$, take from $\mathcal {R}$ a recorded pair (potentially added to $\mathcal {R}$ a couple of steps before) and ask an ATFQ, i.e. a query not related to the current state, but to a previously visited one (Lines 12–13 in Parenting and JPAL, and 32–33 in JPAL-HA). Asking an ATFQ works exactly as asking a BTFQ in terms of adding an entry to $\mathcal {X}$, with the difference that no action is decided now for execution for the current step. Essentially, recording and asking ATFQs is a simple mechanism of *safe exploration* (something like a safe *ε**-greedy*), because the agent may succeed in bringing in $\mathcal {X}$ potentially good actions that otherwise would never be sampled from $\pi (a\!\mid \! s)$ and presented to the human due to their low probability. This is different from the approach taken in [11] where they *executed* the exploratory action, which could mean a ‘straight suicide’ if the agent was for example in a cell surrounded by three cells of water.

The learning at the end of each step (Line 15 in Parenting and JPAL, and 35 in JPAL-HA) is done by following the Bradley-Terry model [20] for estimating score functions from pairwise preferences. That is, we minimise, using gradient descent, the cross-entropy loss between the predicted preference probabilities

$$\begin{aligned} p[P={1^{\text {st}}}] \!=\frac{\pi \left( a_{t}^{(0)} \!\mid \! s_{t}\right) }{\pi \left( a_{t}^{(0)} \!\mid \! s_{t}\right) +\pi \left( a_{t}^{(1)} \!\mid \! s_{t}\right) }, \ \ p[P={2}^{\text {nd}}]\!=\frac{\pi \left( a_{t}^{(1)} \!\mid \! s_{t}\right) }{\pi \left( a_{t}^{(0)} \!\mid \! s_{t}\right) +\pi \left( a_{t}^{(1)} \!\mid \! s_{t}\right) } \end{aligned}$$

and the preference ground-truth labels *μ*, *1-μ*:

2 $$\begin{aligned} \mathcal {L}_{\pi } = -\!\sum _{(s, a^{(0)}, a^{(1)}, \mu ) \in \mathcal {X}} \mu \log p[P={1^{\text {st}}}] + (1\!-\!\mu ) \log p[P={2}^{\text {nd}}] \end{aligned}$$

We minimise this loss using all samples from the dataset $\mathcal {X}$ in each step. For larger environments than the learning paradigm of this paper, standard techniques, such as mini-batch stochastic gradient descent or potentially assigning more weight to recently-added samples in $\mathcal {X}$, could be used. Such extensions, however, would require a mechanism to preserve the influence of samples related to safety, since under-sampling such samples in the mini-batch selection could increase the risk of unsafe actions.

**Algorithm 3:**
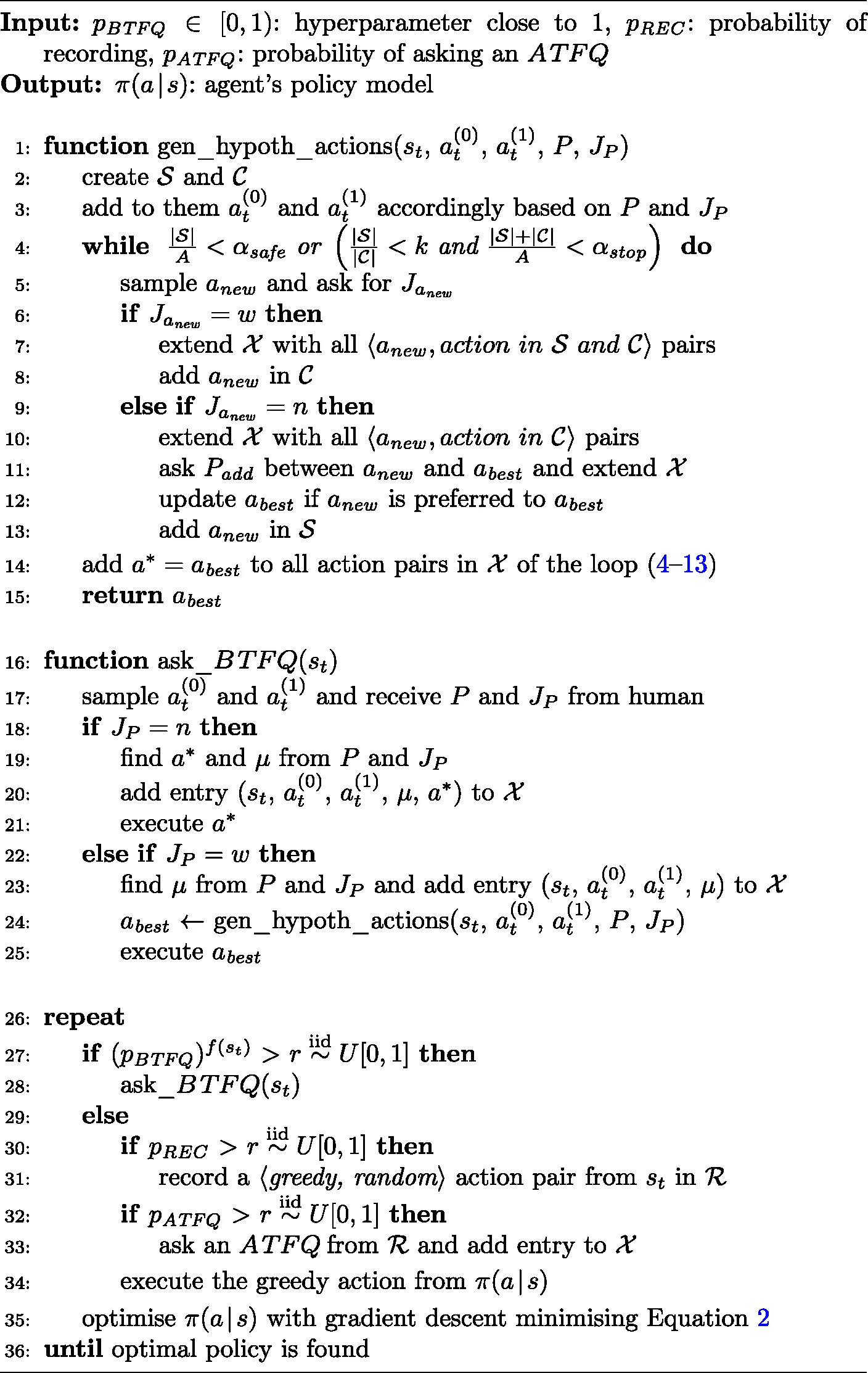
JPAL-HA

At the end of Appendix B, we provide a more complete theoretical explanation of why including safety justifications to preferences can promote safer behaviour than using plain preferences alone. An intuitive explanation is that when the model is uncertain, i.e. when $p[P={1^{\text {st}}}]$ is close to 0.5, the gradient signal of the binary cross-entropy term $p[P={1^{\text {st}}}]-\mu$ has larger magnitude for labels corresponding to safety (e.g. those with *μ =0* or 1) than for labels corresponding to a default reason (e.g. those with *μ =0.25* or 0.75). Thus, safety justifications increase the learning pressure on the policy model to fit the safety-relevant preferences, and as a result they can promote safer behaviour.

## Evaluation

### Experimental setup

To evaluate the impact of justifications and HAs we first tuned and modified Parenting to a great extent so that the comparisons amongst Parenting, JPAL and JPAL-HA were fair. That is, the model architecture, learning rate, learning epochs per step, and the techniques of recording and asking ATFQs were optimised for Parenting and also used for JPAL and JPAL-HA^11^.

For Parenting, JPAL and JPAL-HA, the policy model $\pi (a\!\mid \! s)$ is the average of two components: a CNN for global state representation and a FNN for local information. The CNN processes the board state in an one-hot encoding form, while the FNN uses the type of objects in the four neighbouring cells. More details about the model architecture and training process are provided in Appendix C.1.

Regarding the three hyperparameters of the Parenting, JPAL and JPAL-HA algorithms, we experimented with a *conservative setting*: $p_{{BTFQ}}=0.95$, $p_{{REC}}=0.8$ and $p_{{ATFQ}}=0.8$, and a *relaxed setting*: $p_{{BTFQ}}=0.8$, $p_{{REC}}=0.5$ and $p_{{ATFQ}}=0.5$. The most important parameter is $p_{{BTFQ}}$ defined in Section 3, which essentially trades safety with human burden. A higher $p_{{BTFQ}}$ value increases the number of BTFQs asked to the human, leading to increased user burden, but enhancing safety by reducing the likelihood of the agent taking unsafe actions. Conversely, a lower $p_{{BTFQ}}$ value results in fewer BTFQs, reducing user burden, but increasing the chance of encountering unsafe states. Section 5.4 provides a detailed analysis of the choice of $p_{{BTFQ}}$.

In Sections 5.2–5.7 we run many simulation trials to draw conclusions about the safety and sample efficiency by employing a simulated teacher with the exact behaviour of a rational real human. For the latter, we compute the optimal Q-values at all states of the board and prefer actions with higher Q-values. In Section 5.8, we train a mobile robot with real human users, focusing on the usability and training time of the process. In Section 5.9, we use the results from the real-world experiments to aid us in making a more direct comparison of methods that use different types of human input. Apart from comparing in Section 5.2 with basic traditional RL to demonstrate its inherent limitation in safety during training, our evaluation focuses on comparisons between human-in-the-loop methods because they share the central assumption that the only learning resource is the human. Constrained RL approaches, such as CPO [5], have access to an explicitly specified environmental reward, so comparisons with them would be less direct and interpretable.^12^

### Comparison with traditional RL

**Fig. 4:**
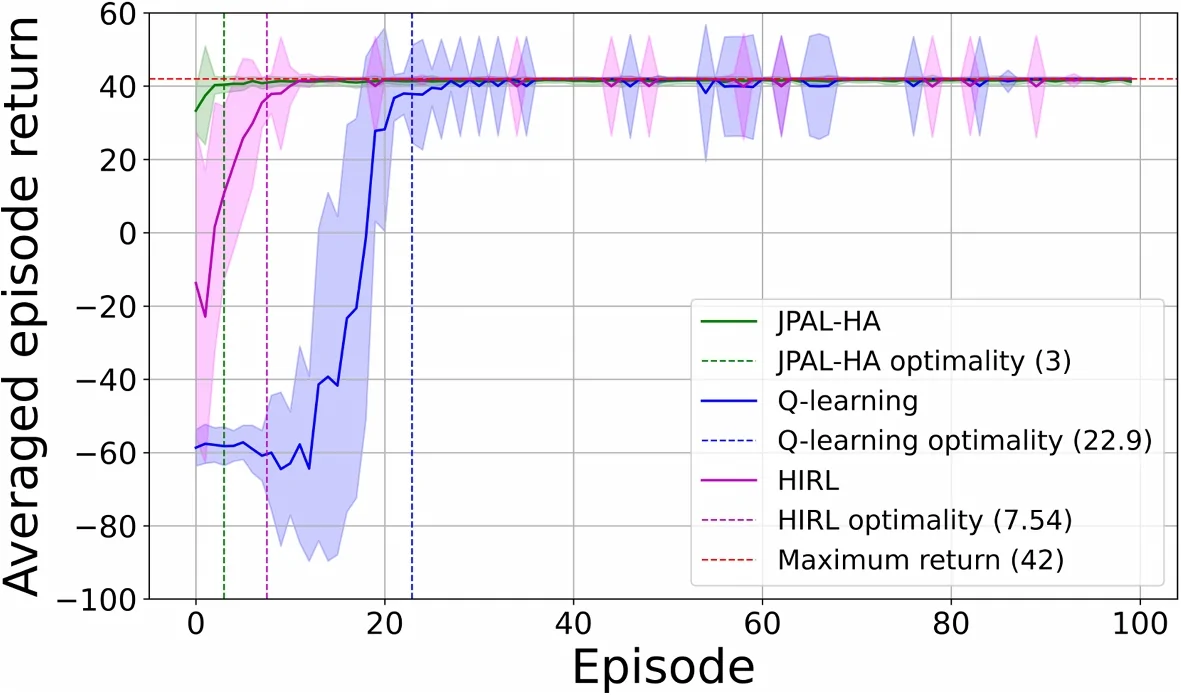
Comparing JPAL-HA with Q-learning and HIRL - Averaged episode return over 50 trials and 1 standard deviation

Initially, we verified our assumption that traditional RL algorithms utterly fail in terms of safety. For Q-learning [21] with *discount \ factor =0.5*, *learning \ rate =1* and *ε* (a small number determining exploration) starting from 0.01 and decaying exponentially^13^, we extracted the blue line of Fig. 4, which illustrates the return for 100 episodes averaged over 50 trials.^14^ The red dashed line depicts the maximum possible return, which is +42 because +50 is the reward for reaching the goal and the agent needs 8 steps to reach it. We found that in 22.9 episodes on average when the optimal policy had been found (i.e. if we deployed the agent, it would give the fastest route), the agent with Q-learning had already suffered *16.54 ± 1.34* deaths^15^, which is considered a very high number, compared to safety-oriented methods. We also experimented with SARSA [23] which is considered ‘safer’ than Q-learning, but still could not drop the number of deaths below 15 which is again by no means satisfactory for safety. In contrast, JPAL-HA with the conservative setting (green line) finds the optimal policy much earlier on average (3 episodes) and suffers only *0.02 ± 0.15* deaths. We realise that with safety-oriented methods, even a single death is typically undesirable. Such rare deaths come when the agent chooses the greedy action from its trained policy model until that step and executes an unsafe action (instead of asking a BTFQ), while it has not mastered the specific state yet (Lines 27, 28 and 34 in Algorithm 3).

Moreover, a subtle point is what would happen if the agent was left training after it could deploy the optimal policy. This is important because in many real-world scenarios, optimality cannot be observed automatically such as in simulations, where we can quickly check after each episode by testing if the return of the updated policy is equal to the maximum possible return or by checking if the actions are optimal while training.^16^ A safe way to observe that the optimal policy has been found in a real scenario would be to deploy and supervise the agent at the end of every single episode. However, that would be very time-consuming. Having an understanding that optimality has almost been reached, we could leave the agent to train more and only then deploy it while supervising it, when we would be more or less certain that we would not have to go through the same time-consuming procedure more than once. As a result, it is important to check that further training would not lead to any potential future deaths. We observe that for JPAL-HA right after the $3^{\text {rd}}$ episode when the agent finds the optimal policy, if we continued training there would be no more deaths.^17^ In contrast, Q-learning would still suffer deaths due to unsafe exploration (we can see that from the spikes after 22.9 on the episode axis). However, we note that the complete absence of further deaths for JPAL-HA was empirically observed for this environment and trials, and is not guaranteed in general, as supervised learning with cross-entropy loss does not always ensure perfect consistency with all previous feedback.

### Comparing Parenting, JPAL and JPAL-HA

Next, we start testing the effect of our novel features, justifications and hypothetical actions. For this section, we make two assumptions: (i) justifications place a marginal additional cost to preferences, which we do not consider; and (ii) extra queries during a loop of hypothetical actions (Lines 4–13 in Algorithm 3) do not impose significant additional burden on the human users as they are concerned with the same state, and do not get bothered repeatedly at regular intervals at those same states. Hence, we wrap everything into a BTFQ. However, from Section 5.6 onwards we do consider and model the additional human time potentially needed.

Table 2 shows the results of simulation experiments acquired over 1000 trials until the optimal policy is found. The Training Deaths row reports their mean number per trial. As an empirical estimate of the probability that the agent visits an unsafe state at least once in a training run, we also report the percentage of trials with at least one death. This is in the conservative setting *6.1%* for Parenting, *3.4%* for JPAL, and *2%* for JPAL-HA; and in the relaxed setting *23.5%*, *17.2%*, and *9.8%*, respectively. When expressed as fractions, these are slightly lower than the corresponding mean deaths because multiple deaths can occur within the same trial; however, they follow the same trend as the mean deaths. We also note that the trend across methods is similar between the two settings (conservative and relaxed) for the different metrics.

**Table 2:** Average number of training deaths, BTFQs, recordings, ATFQs and overall steps in simulation experiments until the optimal policy is found (1000 trials for each with mean and standard deviation reported)

	Conservative setting			Relaxed setting		
	Parenting	JPAL	JPAL-HA	Parenting	JPAL	JPAL-HA
Deaths	*0.07 ± 0.27*	$\mathbf {0.04} \pm 0.23$	$\mathbf {0.02} \pm 0.15$	*0.31 ± 0.61*	$\mathbf {0.22} \pm 0.54$	$\mathbf {0.13} \pm 0.35$
BTFQs	*26.25 ± 5.8*	*31.12 ± 8.21*	*25.92 ± 6.53*	*24.67 ± 5.54*	*29.63 ± 7.54*	*24.02 ± 5.98*
Recordings	*1.59 ± 1.45*	*1.97 ± 1.72*	*1.76 ± 1.64*	*3.85 ± 2.48*	*4.79 ± 3.09*	*4.13 ± 2.86*
ATFQs	*1.32 ± 1.32*	*1.63 ± 1.54*	*1.47 ± 1.45*	*2.61 ± 1.94*	*3.3 ± 2.36*	*2.74 ± 2.13*
Overall steps	*37.72 ± 14.25*	*41.94 ± 15.58*	*37.76 ± 14.5*	*39.86 ± 15.45*	*46.38 ± 17.48*	*40.51 ± 15.59*

**Table 3:** Dunn’s test (*p*-values of pairwise comparisons in each setting)

	Conservative setting			Relaxed setting		
	Training deaths	BTFQs	Overall steps	Training deaths	BTFQs	Overall steps
Parenting/JPAL	$<10^{-2}$	0	$<10^{-8}$	$<10^{-3}$	0	$<10^{-17}$
JPAL/JPAL-HA	0.1	0	$<10^{-9}$	$<10^{-4}$	0	$<10^{-15}$
Parenting/JPAL-HA	$<10^{-5}$	0.28	0.73	$<10^{-15}$	0.02	0.5

Initially, we compare Parenting with JPAL (i.e. using only justifications). For both the conservative and the relaxed settings, we observe an important decrease in the number of training deaths^18^ (safety improved due to justifications), but an increase in BTFQs^19^, which represents extra human burden. We interpret the fewer BTFQs of Parenting in the following way: in Parenting the agent goes through the same ‘unsafe’ routes leading to more deaths while not asking the human many questions. As the familiarity *f*(*s*) increases, the agent acts more and more independently. Thus, we conclude that although the BTFQs increase in JPAL, there is a good reason: safety.

Comparing JPAL with JPAL-HA (adding hypothetical action queries), we observe a further improvement in safety (more significant in the relaxed setting) and a significant decrease in burden (BTFQs). This is happening because the agent asks many queries in one go when it is in a ‘dangerous’ area and the human does not need to be bothered repeatedly with such cases. We believe that in most cases it is reasonable to assume that asking a lot of queries about the same state at once is not as time-consuming for the human as multiple bothering BTFQs; nobody enjoys being repeatedly interrupted, whereas quickly answering a batch of related questions is less disruptive^20^. As noted in Section 4.2, most of these hypothetical action queries (asked until a second safe action is found) do not require a preference answer *P*, but simply a single justification over a new (hypothetical) action $J_{a_{{new}}}$ (i.e. ‘if I had taken that other action, would it have been safe?’).

We conducted significance tests for the variables of training deaths, BTFQs and Overall steps to observe if there is a statistical difference amongst Parenting, JPAL and JPAL-HA. We used the significance level *α =0.05*. Initially, we verified that the assumptions of homoscedasticity (equal variances) and normality (data generated from the normal distribution) did not hold for our data. This was done, for both conservative and relaxed settings, by inspecting (i) the boxplots which confirmed different spreads amongst the three methods (which is consistent with the different standard deviations in Table 2), and (ii) the histograms which indicated right-skewed distributions for most variables. Thus, we conducted the non-parametric Kruskal–Wallis one-way analysis of variance test [24] (which does not make these assumptions) to determine if there is a statistical difference for the means amongst the three different methods. As expected, *p*-values for all variables and for both the conservative and relaxed setting were much smaller than *α*, so we conclude that there is a clear significant difference amongst the three methods. The interesting question was to check the pairwise differences between methods, for which we conducted the post-hoc, non-parametric pairwise Dunn’s test [25]. We also needed to tackle the multiple comparisons problem (due to three pairwise hypothesis tests) by controlling in our case the familywise error rate (FWER) (i.e. we wish to minimise the false positives) instead of the false discovery rate (which gives better power, i.e. caring about a better true positive rate). The correct adjustment in this case is the Holm-Bonferroni [26], which dominates the plain Bonferroni correction and is used under arbitrary assumptions. Table 3 shows the adjusted *p*-values which we compare with *α =0.05*. With reference to Table 2, in terms of training deaths, we observe a significant difference with the introduction of JPAL for both the conservative and relaxed setting and a significant difference with the introduction of hypothetical actions in the relaxed setting (a reduction of training deaths). In terms of BTFQs and overall steps the unfavourable for JPAL significant difference with Parenting (BTFQs and overall steps increased), is fixed with the introduction of JPAL-HA (favourable for JPAL-HA significant difference with JPAL — BTFQs and overall steps dropped), where in the relaxed setting we even observe a significant difference in favour of JPAL-HA compared to the initial Parenting (*p=0.02*). In short, we managed to improve safety without deteriorating BTFQs or overall steps both in the conservative and relaxed setting.

### Analysing the choice of $p_{{BTFQ}}$

Regarding safety, an empirical observation is that increasing $p_{{BTFQ}}$ to a high value such as 0.99, which also avoids continuously bothering the human, almost always yields zero deaths. This means that our method can be useful also for environments with a low tolerance for unsafe events, where it would be appropriate to choose a higher $p_{{BTFQ}}$ (possibly higher than 0.99). In contrast, in less dangerous cases, such as washing the dishes, we would not mind relaxing $p_{{BTFQ}}$ for the sake of less human time and potentially sacrificing a few plates.

**Fig. 5:**
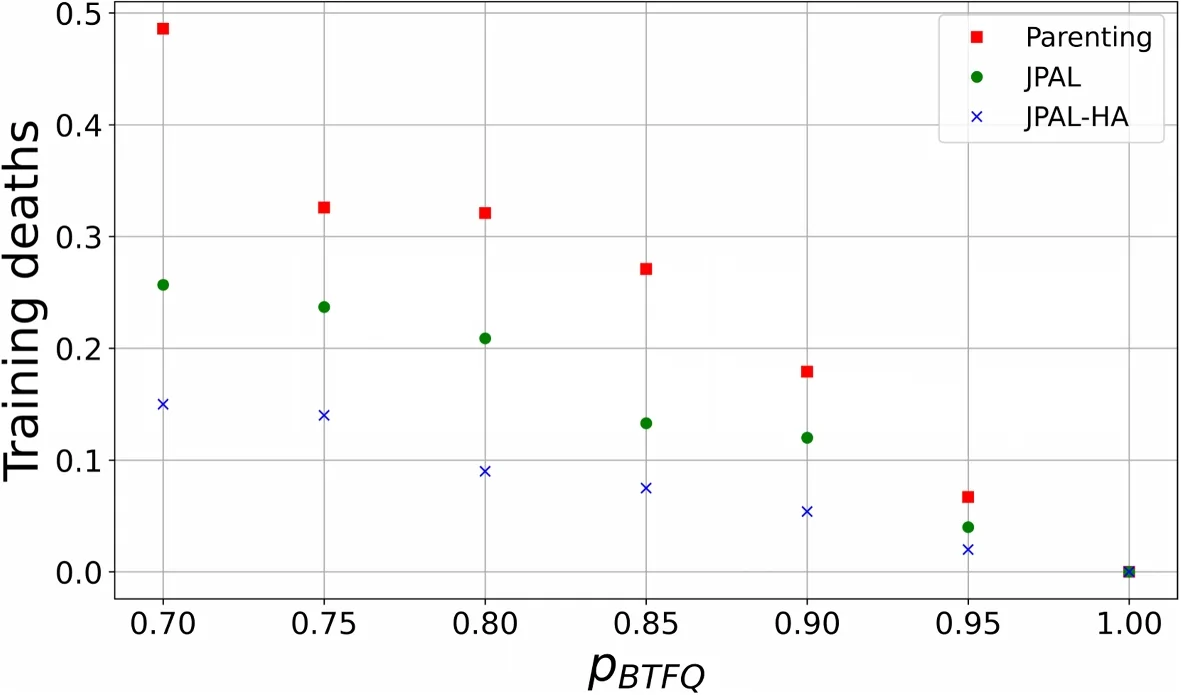
Training deaths versus $p_{{BTFQ}}$

**Fig. 6:**
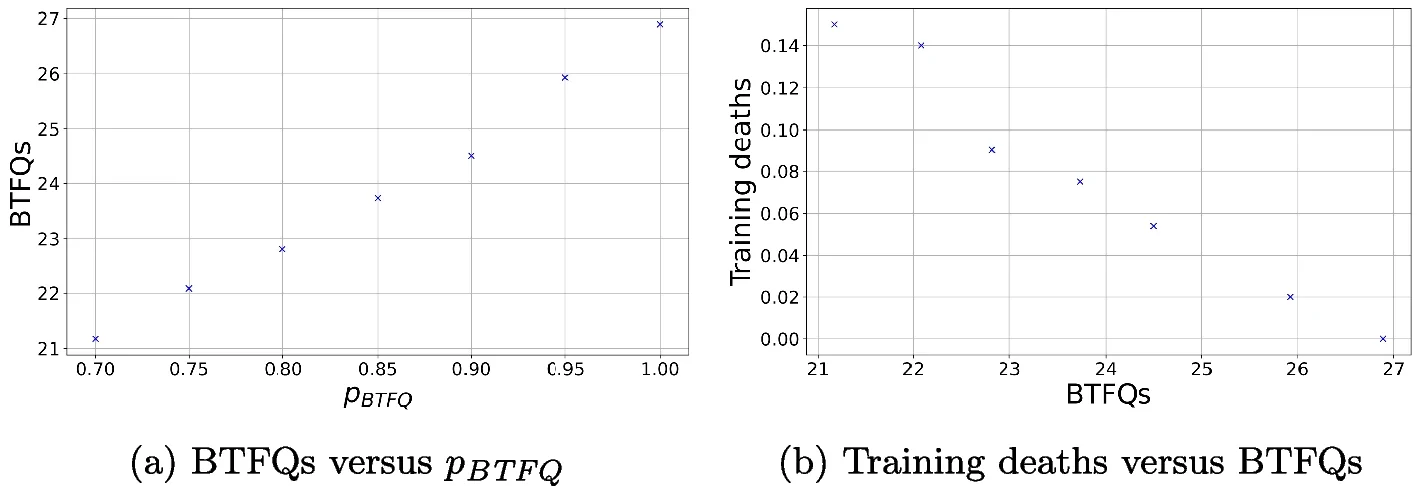
JPAL-HA safety/human burden trade-off

Figure 5 summarises the evaluation of Parenting, JPAL and JPAL-HA in terms of safety (mean values extracted over 1000 trials). We keep constant the hyperparameters $p_{{REC}}=0.8$ and $p_{{ATFQ}}=0.8$ and we alter the most important one $p_{{BTFQ}}$. Firstly, if we compare the three methods at any $p_{{BTFQ}}$ point (except for $p_{{BTFQ}}=1$ which, although yielding zero deaths, would normally be undesirable due to constant supervision), we observe that JPAL is significantly safer than Parenting, and JPAL-HA is safer than JPAL (the latter is more distinct in more ‘relaxed settings’ ($p_{{BTFQ}}\le 0.9$)). Moreover, we observe that as we increase $p_{{BTFQ}}$, all methods improve safety until they reach zero deaths. This indicates the safety/human burden trade-off discussed earlier. To make it more evident, if we plot the BTFQs versus $p_{{BTFQ}}$ curve for JPAL-HA we observe the almost linear ascending relationship of Fig. 6a (similar for Parenting and JPAL). Hence, as expected, Figure 6b, which plots the training deaths versus BTFQs curve (real trade-off) for JPAL-HA, has almost the same trend as Fig. 5 and clarifies the trade-off between safety (training deaths) and human burden (BTFQs).

### Recording and asking ATFQs

As described in Section 4.3, recording and asking After-The-Fact-Queries is an additional mechanism for safe exploration. We noticed in some trials that when the agent was becoming very ‘confident’ from a limited number of actions in a state and would not sample other better actions to present them for a query to the user, an ATFQ could help it escape a potential behavioural loop (i.e. by going back and forth between two neighbouring states) learnt by chance^21^.

Another benefit of ATFQs is characteristic to relaxed settings (lower $p_{{BTFQ}}$ values). A relaxed setting is used when the human does not wish to be bothered consecutively by a lot of BTFQs, which need to be answered on the spot. Since ATFQs are queries about states visited in the past and recorded in the temporary memory, they do not need to be necessarily answered there and then, as they will not affect safety at that step (nothing will change if they are answered a few steps later). Indeed, as we can see in Table 2, although the aggregate of queries (BTFQs and ATFQs) is almost the same for both the conservative and relaxed setting, the additional ATFQs in the latter setting give extra ‘relaxation’ to the user to answer at their convenience.

### Unwrapping a JPAL-HA query

Even though in Section 5.3 we found no significant difference in terms of BTFQs between Parenting and JPAL-HA, this was based on the assumption that justifications and queries following the hypothetical actions loop place marginal additional burden, which we did not consider. The idea for that was that a user puts effort to collectively study what happens in a state from where queries are issued, rather than they spend equal time for each different query from the same state. Hence, in a collective preference and justification query, the additional burden to answer the justification would be marginal, as well as the burden of additional queries during a hypothetical actions loop. These assumptions would be more appropriate if for the human burden we also considered the cognitive load, instead of only the number of different queries or the input time. Thus, although the above assumptions may be reasonable in some cases, we do not consider them as the general case, and we also present evaluation without them. We start here by ‘unwrapping’ the different types of queries, and then evaluate further in Sections 5.8 and 5.9.

In JPAL-HA a query can take one of the following types:

1. $P^{({BTFQ})}$: preference between initial $\langle a_{t}^{(0)}$, $a_{t}^{(1)} \rangle$ from a BTFQ
2. $P^{({ATFQ})}$: preference between initial $\langle a_{t}^{(0)}$, $a_{t}^{(1)} \rangle$ from an ATFQ
3. $P_{{add}}^{({BTFQ})}$: additional preference between $\langle a_{{new}}$, $a_{{best}} \rangle$ from a BTFQ
4. $P_{{add}}^{({ATFQ})}$: additional preference between $\langle a_{{new}}$, $a_{{best}} \rangle$ from an ATFQ
5. $P_{{add}}^{({DP})}$: additional preference between $\langle a_{{new}}$, $a_{{best}} \rangle$ from a *DP*
6. $$J_{P}^{({BTFQ})}$$: justification over $$P^{({BTFQ})}$$
7. $$J_{P}^{({ATFQ})}$$: justification over $$P^{({ATFQ})}$$
8. $J_{a_{{new}}}^{({BTFQ})}$: justification over $a_{{new}}$ from a BTFQ
9. $J_{a_{{new}}}^{({ATFQ})}$: justification over $a_{{new}}$ from an ATFQ
10. $J_{a_{{new}}}^{({DP})}$: justification over $a_{{new}}$ from a *DP*

We assume that the human needs more cognitive effort to answer preference queries (1–5) than justification queries (6–10). Moreover, for simplicity we assume that justifications over two actions (6–7) require the same effort as justifications over a new (hypothetical) action (8–10) — this will be tested more thoroughly in Sections 5.8 and 5.9. Parenting includes only query types 1 and 2, and JPAL includes queries 1, 2, 6 and 7. Table 4 shows the breakdown of different query types, with the values acquired in the conservative setting.

**Table 4:**
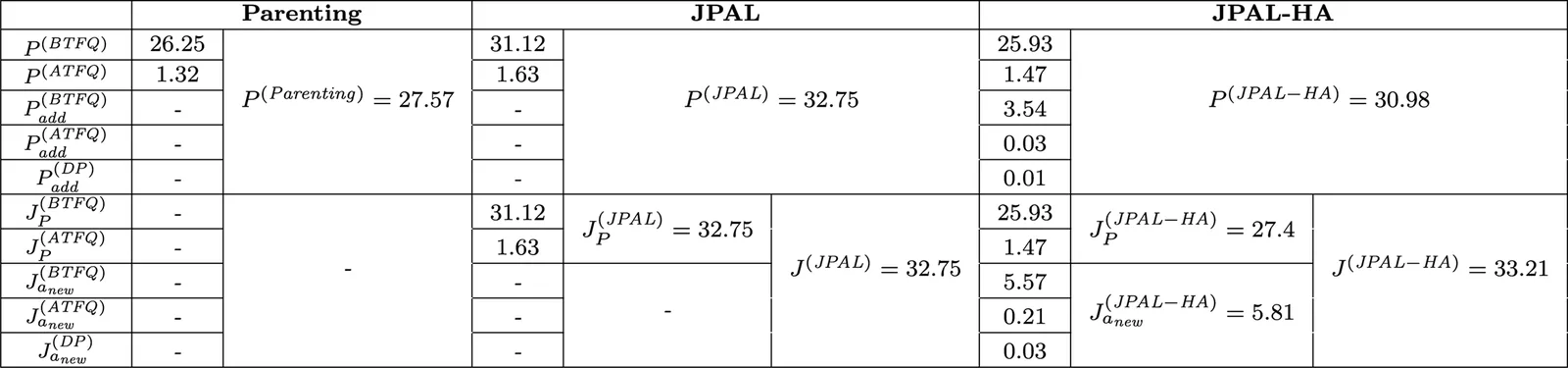
Comparing Parenting, JPAL and JPAL-HA with all types of queries

Parenting takes the least time as it requires the fewest preference queries to be answered and does not seek justifications. However, we have already seen that JPAL significantly improves safety during learning and JPAL-HA also offers additional improvements in safety (Fig. 5). Comparing JPAL and JPAL-HA with respect to time required to respond to queries, although the number of justification queries issued by JPAL-HA, $J^{({JPAL-HA})}$, is very slightly higher than that by JPAL, $J^{({JPAL})}$, JPAL-HA issues fewer preference queries, i.e. $P^{({JPAL-HA})}<P^{({JPAL})}$. This reduction in preference queries is observed in all settings, indicating that hypothetical action queries reduce the need for more preference queries. Given also that when using HAs the overall learning steps decrease in comparison to using only justifications (Table 2), we hypothesise that HAs help to decrease the overall human burden. The hypothesis $t^{({Parenting})}<t^{({JPAL-HA})}<t^{({JPAL})}$ is tested in Section 5.8 through experiments with people.

### Queries or human intervention?

Before we present the evaluation of Parenting, JPAL and JPAL-HA with real human trainers and a robot in terms of actual training time, we compare our approach with a different class of human-in-the-loop safe learning methods that rely on the human teacher to intervene when an unsafe action is about to be taken, rather than the agent querying the human before taking an action. We compare JPAL-HA as closely as possible with Human Intervention RL (HIRL) [22]. We did not compare with ReQueST [17, 27], another human method focusing on safety, because the necessary approximations due to different assumptions would make a comparison unrealistic; for example, ReQueST uses off-policy data raising the question of how these data can be collected.

HIRL [22] learns in three stages: (i) the agent learns a policy using standard RL methods with a human overseeing it, blocking catastrophic actions and proposing an alternative safe (normally the optimal one when they understand the environment well^22^) action; (ii) the agent pauses and a binary catastrophe classifier is built from the record of interventions from Stage (i) using supervised learning; and (iii) the agent starts again, but now the human is substituted by the catastrophe classifier (a kind of governor). More details and the experimental setup for HIRL are given in Appendix C.2.

**Fig. 7:**
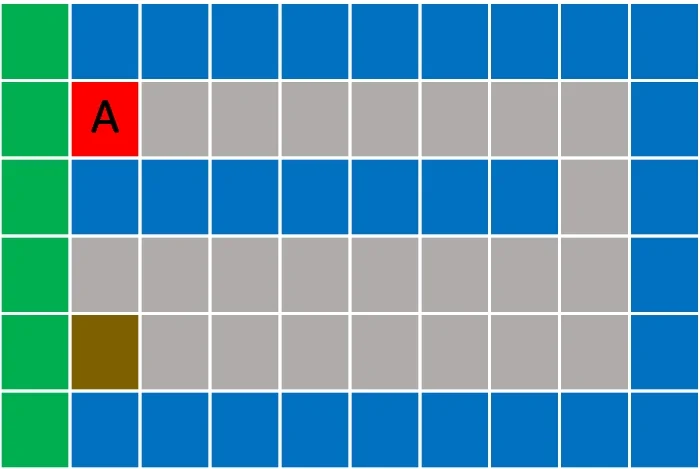
Island Navigation environment configuration with more dangerous states

**Fig. 8:**
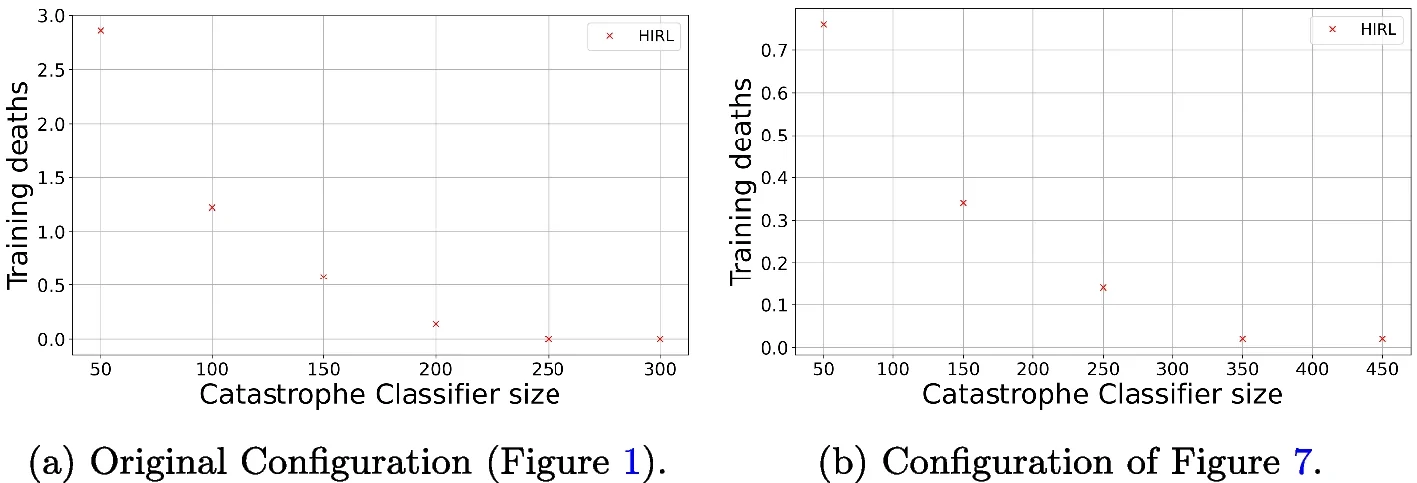
Safety/human burden trade-off in HIRL

The comparison to JPAL-HA relies on the reasonable assumption that a preference *P* plus a justification $J_{p}$ (or $J_{a_{{new}}}$ for simplicity) query with JPAL-HA requires more or less the same time as an *Intervention* (i.e. a block plus a proposal of a new action) in HIRL.^23^ Also, given both algorithms consider the trade-off between safety and human burden, we use safety as the top priority; i.e. minimising training deaths for both. We consider two environment configurations, the original (Fig. 1) and the one illustrated in Fig. 7. JPAL-HA had almost no training deaths in each configuration with the conservative setting, and for HIRL this depends on the steps the human would constantly supervise (or equivalently on the samples used to build the catastrophe classifier — Fig. 8). We then compared the two methods using: (1) how much information needs to be extracted from the human (we compare $P^{({JPAL-HA})}$ and $J^{({JPAL-HA})}$ with ${Interventions}^{({HIRL})}$); and (2) how long it takes to have the human oversee the agent (we compare the overall steps until optimality in JPAL-HA ${Steps}^{({JPAL-HA})}$ with the overall steps until we can release the human in HIRL ${Steps}^{({HIRL})}$).

In the original configuration (Fig. 1), for JPAL-HA we reproduce the results for $P^{({JPAL-HA})}$, $J^{({JPAL-HA})}$ and ${Steps}^{({JPAL-HA})}$ from earlier in Table 5. For HIRL, we ran experiments to determine how many steps a human trainer needs to monitor the agent and intervene as necessary to achieve a similar number of training deaths on average. From Fig. 8a, we find that we need the human trainer to monitor for approximately 250 steps. While the algorithm continues with the binary classifier and will take an average of 291.64 steps to find the optimal policy, we assume that the time the agent operates by itself is not included in the human burden. In Fig. 4, these 291.64 steps correspond to 7.54 episodes (magenta dashed line), where we observe again the superiority of JPAL-HA in terms of the overall training time. Across the 250 supervision steps, the human trainer had to intervene an average of 16.12 times. Thus, HIRL needs to extract less information from the human trainer. However, JPAL-HA is far better in terms of the time the human has to ‘wait in their chair’. Moreover, with JPAL-HA the human trainer can focus on other tasks with the agent being smart enough to ask the human when it predicts that it needs more information. Instead, with HIRL the human has to constantly monitor the agent and intervene (although the human still may relax if they are certain that the RL agent will be in a safe region for some time). Moreover, if the environment has many hazards, such as in the configuration illustrated in Fig. 7, then JPAL-HA is competitive with respect to information need with $P^{({JPAL-HA})}$ and $J^{({JPAL-HA})}$ much closer to ${Interventions}^{({HIRL})}$ (Table 5). JPAL-HA continues to dominate HIRL with respect to time demands on the human trainer in this scenario. Moreover, the optimal policy (even if using the human only for the first 350 steps) was found after 533.92 steps on average, which illustrates its sample inefficiency.

**Table 5:**
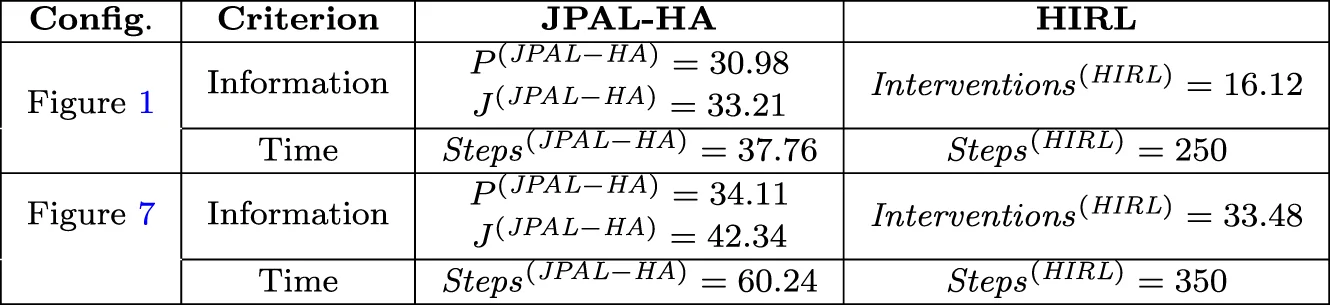
Information required from, and time commitment of human trainer for JPAL-HA and HIRL for two environment configurations

### User experiments

**Fig. 9:**
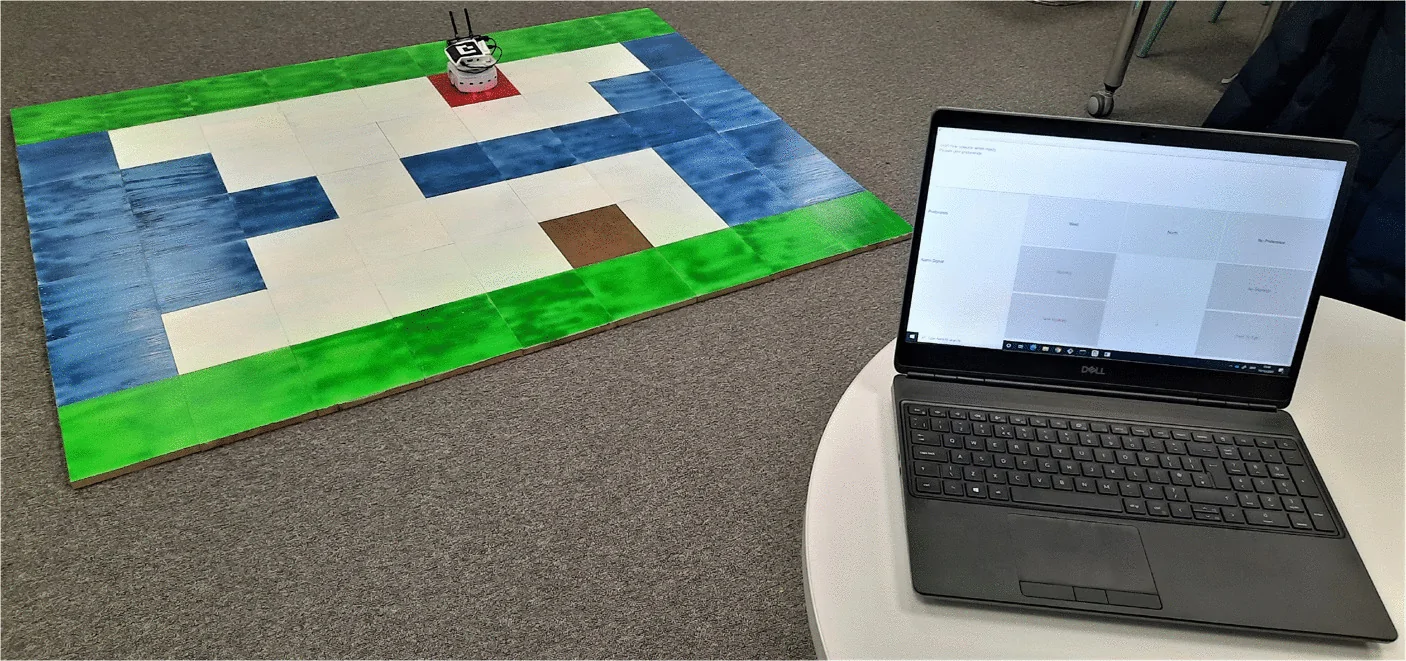
Experiment configuration with a Thymio mobile robot

**Fig. 10:**
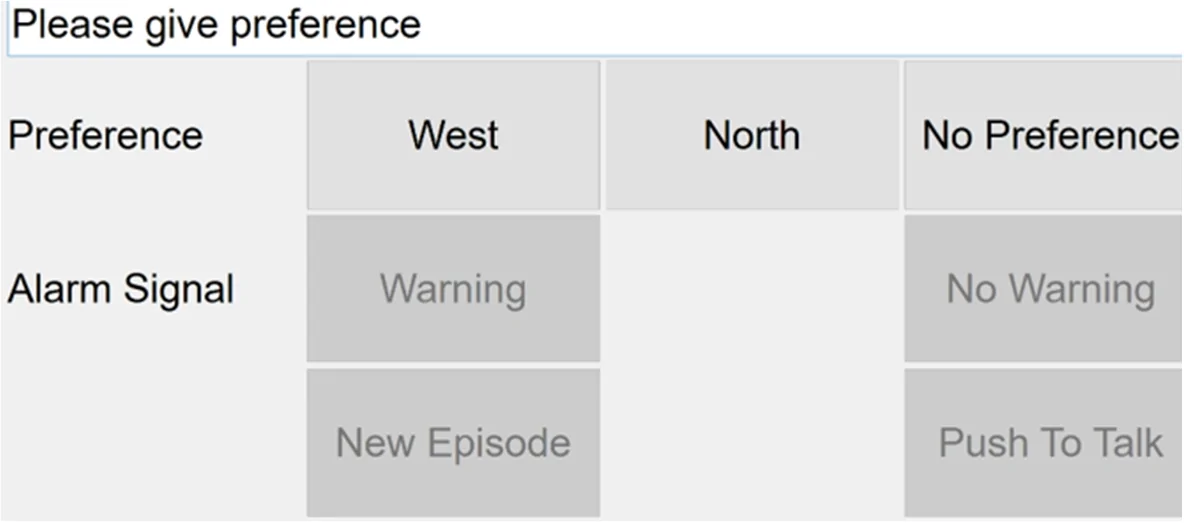
Instance of the graphical interface the participants used to interact with the robot in JPAL and JPAL-HA. *Alarm Signal* is equivalent to justifications. With *Push To Talk* participants could give their answer through a microphone; however, we did not use this button during the experiments

In order to fully understand the impact of our approach, we conducted experiments with people answering queries to train a Thymio mobile robot [28]. The configuration of Fig. 1 (the same as the one of the simulation experiments) was created with wooden pieces (Fig. 9). The participants used a computer next to the arena to answer preference and justification queries using a graphical interface (Fig. 10). The answers were relayed to a Raspberry Pi attached above Thymio that controlled its movements. Thymio executed the actions at an average speed of 20 cm/s. ATFQs were presented on the laptop screen. After the execution of each episode, Thymio was placed at the initial state manually.

Our aim is to answer two questions: (i) Can training using human preferences and justifications be completed in a reasonable amount of time? And (ii) does our hypothesis from Section 5.6 that $t^{({Parenting})}<t^{({JPAL-HA})}<t^{({JPAL})}$ hold? To answer these questions, we needed for each of the three methods (Parenting, JPAL, and JPAL-HA) to identify and run with all participants a specific trial that returned average values for all the variables involved during training (i.e. training deaths, overall steps/episodes, number of preference/justification queries from BTFQs/ATFQs, recordings, etc.). We first obtained these average values from the 1000 simulation trials at the conservative setting, reported in Tables 2 and 4. Then we ran multiple trials in simulation until we identified a specific one for each method, where the different end metrics closely matched these average values. For example, we chose trials with zero training deaths and three episodes until optimality for all Parenting, JPAL and JPAL-HA. We stored the seed of the identified trials and used those with all participants for fair and standardised comparisons.^24^ Consequently, the primary metric we focus on in this section is the real training time it takes the participants to complete the specified trials with each method, while these experiments do not constitute evidence of safety.

**Fig. 11:**
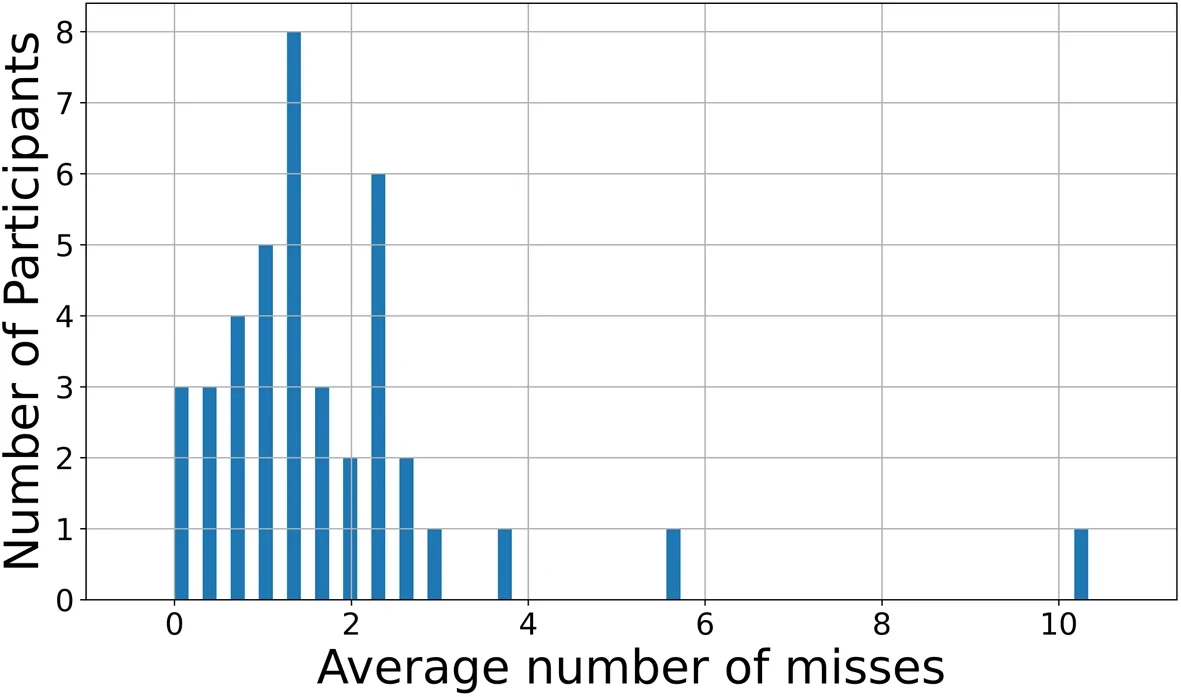
Histogram of the average number of misses across all three methods (Parenting, JPAL and JPAL-HA). A miss corresponds to an incorrect response to either a preference or justification query, typically caused by inattention. The two extreme points on the right were excluded from the results as outliers

We recruited 40 participants^25^ overall for around one hour each, paying for their time. They consisted mainly of university students, but also included university staff and members of the general public. 65% of participants had backgrounds related to computer science, but in the other 35% we had a good diversity in terms of discipline, such as linguistics, medicine, engineering, law, administration, etc. The gender balance was 13 female and 27 male. The experiments were separated into two groups, with 18 participants in the first and 22 in the second. For each participant, we devoted around 20 minutes for them to practise in a smaller configuration to get used to interacting with the robot through the interface. All participants were asked whether they were comfortable interacting with the robot. We recorded ‘misses’ (i.e. giving a suboptimal or bad answer to a query) for all participants, and excluded two outliers from our analysis (Fig. 11). We did this because our aim was to focus on training time rather than training with human input that varies in trustworthiness; cf. [29]. Although some ‘misses’ were to be expected by oversight or rush during the experiments, enforcing an average per participant to be within a low mean and tight distribution was, we believe, reasonable.

**Fig. 12:**
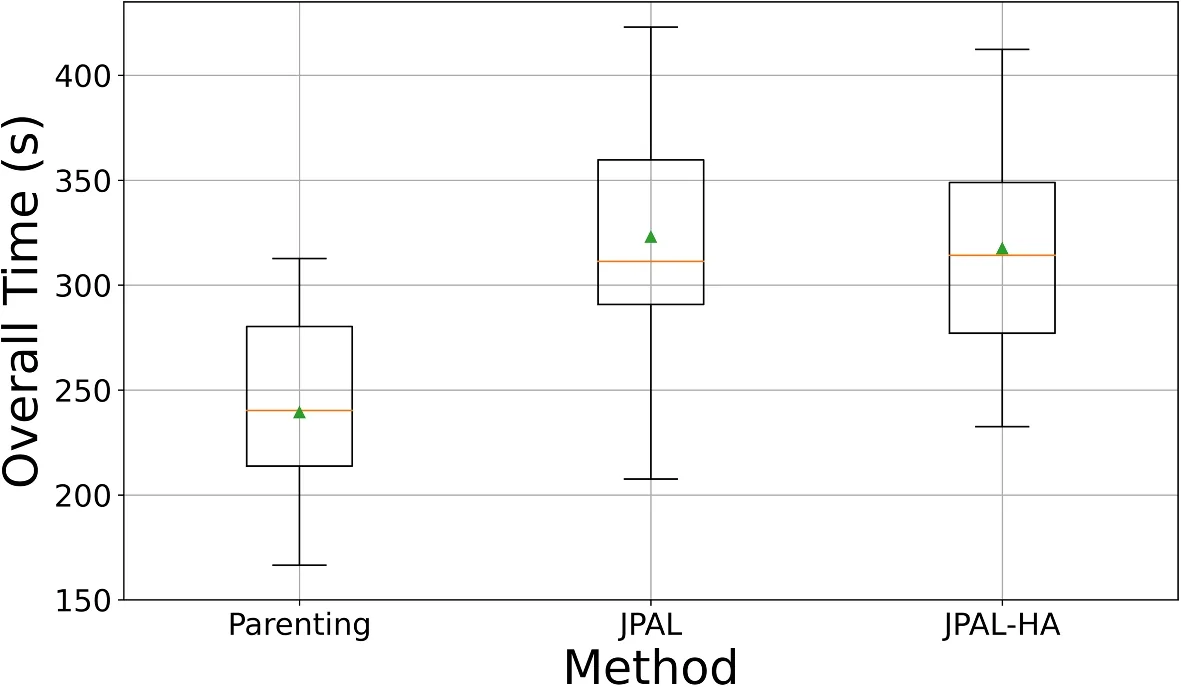
Overall training time for the first group of participants

Although we provided initial training with the smaller configuration, there was a further training effect where participants tended to perform better in later trials. For the **first** group of participants, we reduced this training effect by permuting the order of the three methods and changing the configuration by rotating it 90 or 180 degrees to avoid memorisation. The participants ran each method once, and the boxplot of each method is shown in Fig. 12. These results indicate that training with each of the three methods can be completed in a reasonable time (slightly more than 5 minutes with JPAL and JPAL-HA).

To determine whether there are statistically significant differences amongst the three methods, we initially observed, again as in the simulation experiments, the boxplots and histograms of the data. Although it seemed that the assumptions of homoscedasticity and normality might hold, we decided not to rely on them since our sample size was moderate, opting instead to run again non-parametric tests which are robust in all cases. The non-parametric Kruskal–Wallis test indicated a significant difference amongst the three methods. Using Dunn’s test, with the Holm-Bonferroni adjustment of the *p*-value, there is a significant difference between Parenting and the other two methods, but no significant difference between JPAL and JPAL-HA (Table 7). Although there is a significant difference between JPAL-HA and Parenting, this is around 30% on average. Further investigation into these results revealed that a significant training effect remained due to the extra features of JPAL and especially of JPAL-HA that the participants had to get used to; participants almost always took less time using the second method, regardless of whether this was JPAL or JPAL-HA.

**Table 7:** Adjusted *p*-values from Dunn’s test for the first group and from the Mann-Whitney U (Wilcoxon rank-sum) test for the second group

Comparison	First group	Second group
Parenting/JPAL	0.001	-
JPAL/JPAL-HA	0.910	0.449
Parenting/JPAL-HA	0.001	-

**Fig. 13:**
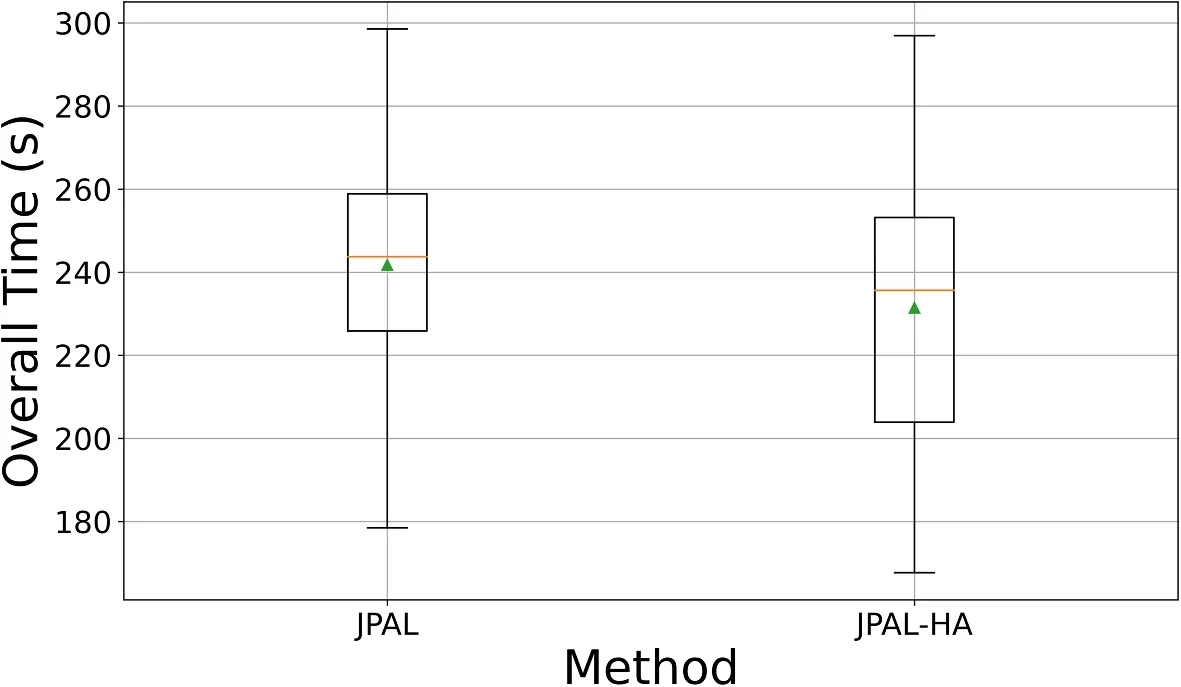
Overall training time for the second group of participants for their second use of JPAL and JPAL-HA

In order to investigate this further, we recruited the **second** group of participants, and, to reduce the training effect, we asked participants to run JPAL and JPAL-HA twice. This modification was made to ensure that the training effect would not skew the results and to provide a fair comparison between JPAL and JPAL-HA. The participants also started with Parenting to give additional practice. The participants then ran JPAL and JPAL-HA in different orders and by rotating the configuration. The results again showed subtle differences between them on the first try. However, on the second try, we found $t_{{JPAL}}=242 \pm 33.3$ and $t_{{JPAL-HA}}=231 \pm 34.9$ (Fig. 13); i.e. participants took slightly less time on average using JPAL-HA than JPAL (approximately 11s). Similarly to the first group, we decided to follow a non-parametric test. Analysing the results from these trials, there is no significant difference (Mann-Whitney U (Wilcoxon rank-sum) test [30]) between JPAL and JPAL-HA, but we do observe a shift in the *p*-value from the first group (Table 7). Looking in depth at the data for these experiments, we observed that the average response time for $J_{a_{{new}}}$ queries (2.45s) (i.e. justifications over hypothetical actions in JPAL-HA) was significantly greater than for $J_{P}$ queries (1.15s) (i.e. justifications over preferences used in both JPAL and JPAL-HA). Our explanation for this is that $J_{a_{{new}}}$ queries come sparsely and users do not expect them in every step like $J_{P}$ queries, and that $J_{a_{{new}}}$ queries refer to a new action that the users have not yet considered. This may be part of the explanation that we do not see a significant difference between JPAL and JPAL-HA with human participants.

Most importantly, the time required to train the agent using both JPAL and JPAL-HA is now significantly reduced from the average of the first group of participants (from around 5 minutes to around 4 minutes), which is close to the average for Parenting in the first group. It is also very close to the average for Parenting in the first group, even when only the participants who ran Parenting third in the row are considered (meaning they already had significant practice after running JPAL and JPAL-HA). The latter’s average time was 219s, which is only 12s less than $t_{{JPAL-HA}}=231$. This means that, even without the assumption that justifications and hypothetical action queries are a marginal cost, the training time can be kept with JPAL and especially JPAL-HA to admissible levels, not very far from the one of Parenting.

To summarise our findings, when given initial practice, the participants took around 30% longer to train the Thymio robot using either JPAL or JPAL-HA than for Parenting. When participants had additional training in providing justifications and answering hypothetical action queries, however, the training times were very similar. Our hypothesis, $t^{({Parenting})}<t^{({JPAL-HA})}<t^{({JPAL})}$ is not supported, but it is reasonable to conclude that $t^{({Parenting})}\simeq t^{({JPAL-HA})}\simeq t^{({JPAL})}$. Thus, the time required to train the agent is similar for people from a diverse set of backgrounds, but we have significant added benefits with respect to safety during the learning process.

### Comparisons by human input and training time

**Fig. 14:**
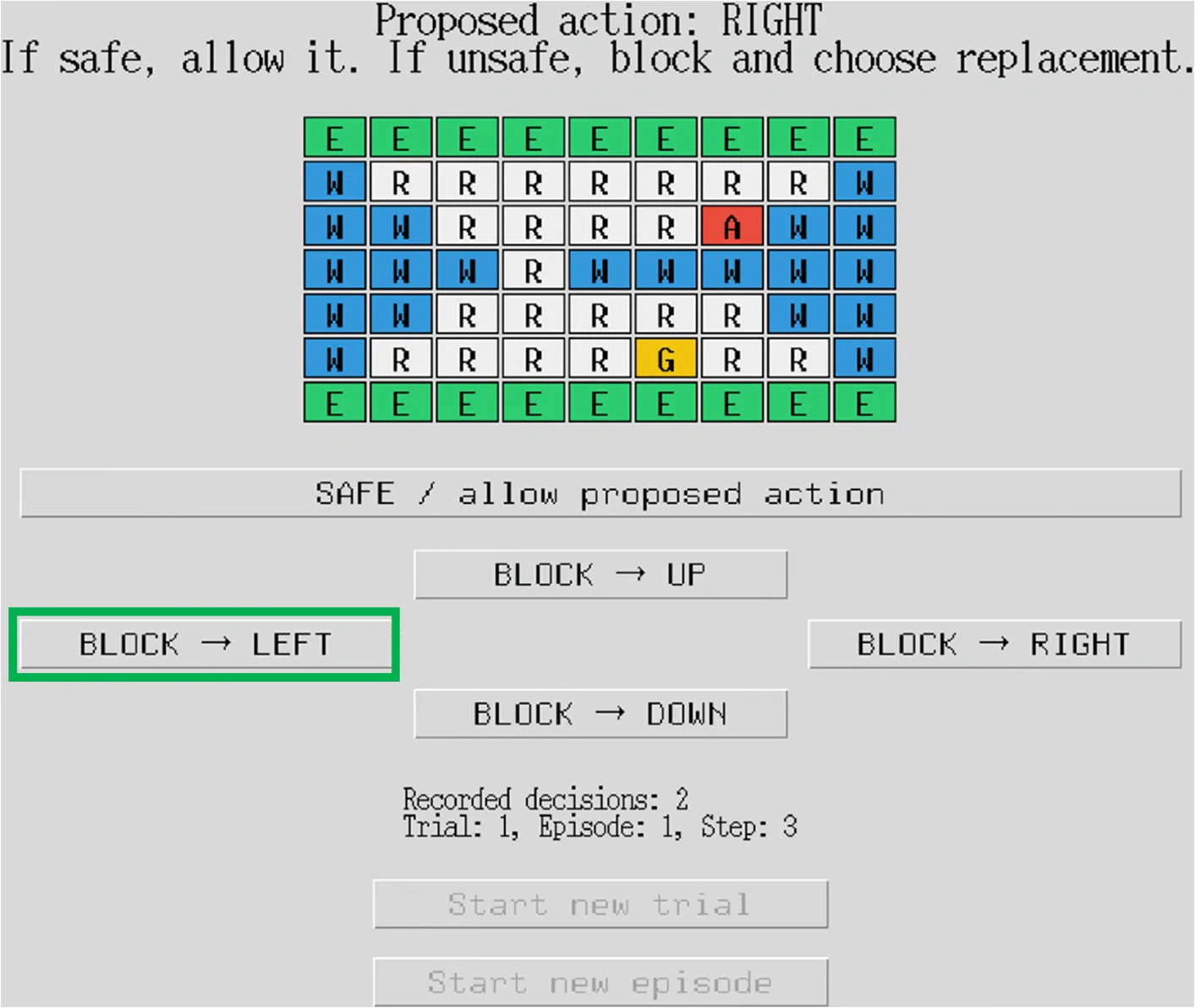
GUI of HIRL. In the instance shown the agent proposes (gets ready to move) RIGHT, so the user blocks that action and chooses LEFT which leads faster to the goal

From the experiments with real users of Section 5.8 we can obtain the average response time across all responses and labellers for the different types of queries. These are for a preference $t_P=3.83s$, for a justification over a preference $t_{J_{P}}=1.15s$ and for a justification over a hypothetical action $t_{J_{a_{{new}}}}=2.45s$. With this information we can directly compare Parenting, JPAL and JPAL-HA in terms of human feedback time. For example, using the mean numbers of different types of queries from Table 4, the mean human input time until the optimal policy is found for JPAL-HA is calculated by: $t_{P} \times P^{(JPAL-HA)} + t_{J_P} \times J_{P}^{(JPAL-HA)} + t_{J_{a_{{new}}}} \times J_{a_{{new}}}^{(JPAL-HA)}$. We can also include in this comparison HIRL. Although we did not run a full study with many participants, one of the authors provided around 1200 answers (running two trials from the original Island Navigation configuration and the one of Fig. 7) based on the GUI shown in Fig. 14. We separated the response time of queries in three categories: (i) those asked far from a water cell where the user always allows the proposed action (1.58s); (ii) those next to a water cell with the proposed action of the agent leading to a safe action that the user allows (1.84s); and (iii) those next to a water cell with the proposed action leading to water, where the user interrupts the agent and dictates the optimal action (3.14s). Given these, we can either include category (i) in our comparisons or consider its time zero (passive supervision). We include category (iii), as well as (ii) since when the agent goes next to a dangerous state the user must be ready to act even if the proposed action is safe. Given the above, similarly to Parenting, JPAL, and JPAL-HA, we can multiply the mean response times of different types of inputs by their mean number until optimality and calculate the mean human feedback time.^26^

**Fig. 15:**
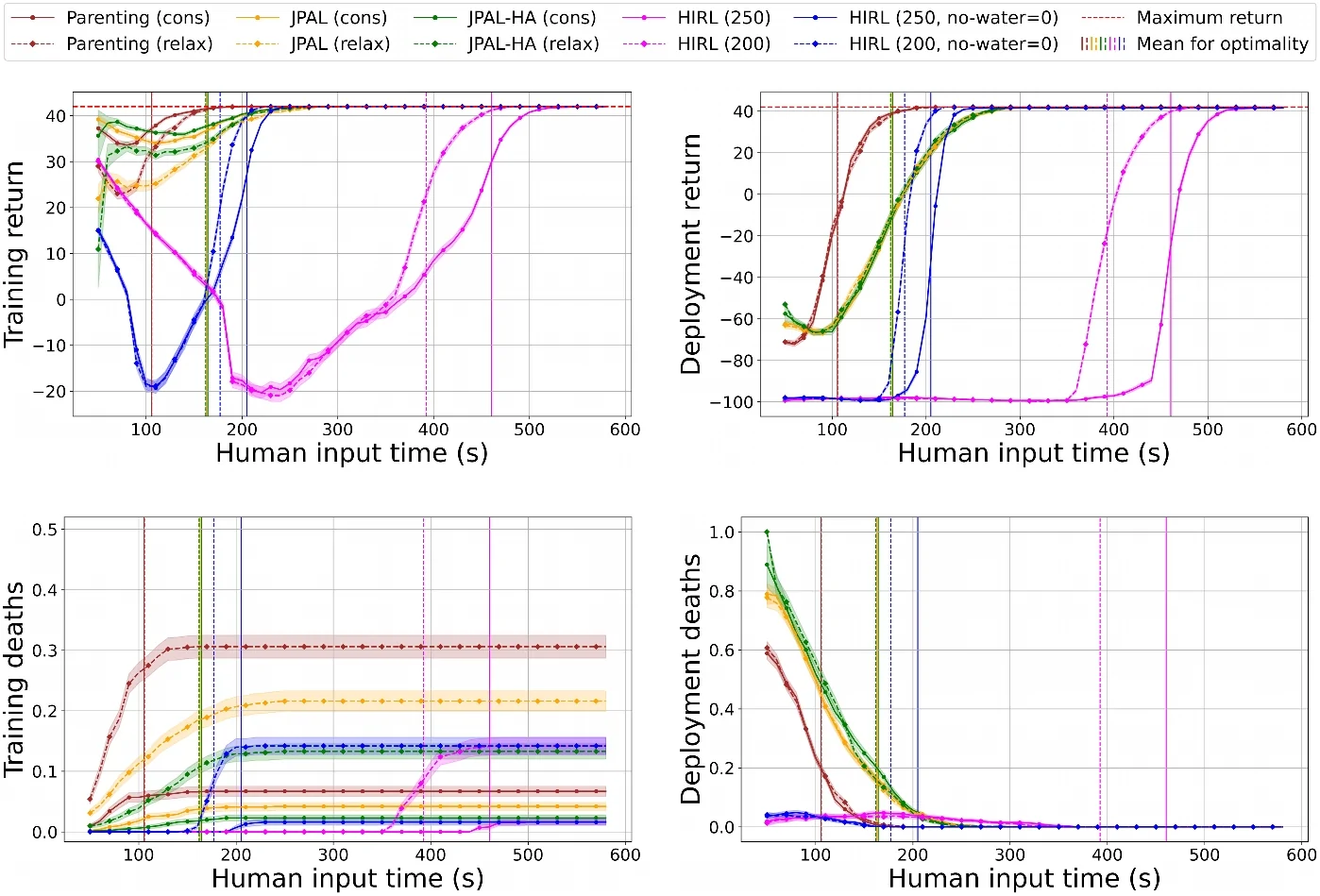
Training/Deployment performance and safety in relation to human input time (Configuration of Figure 1)

**Fig. 16:**
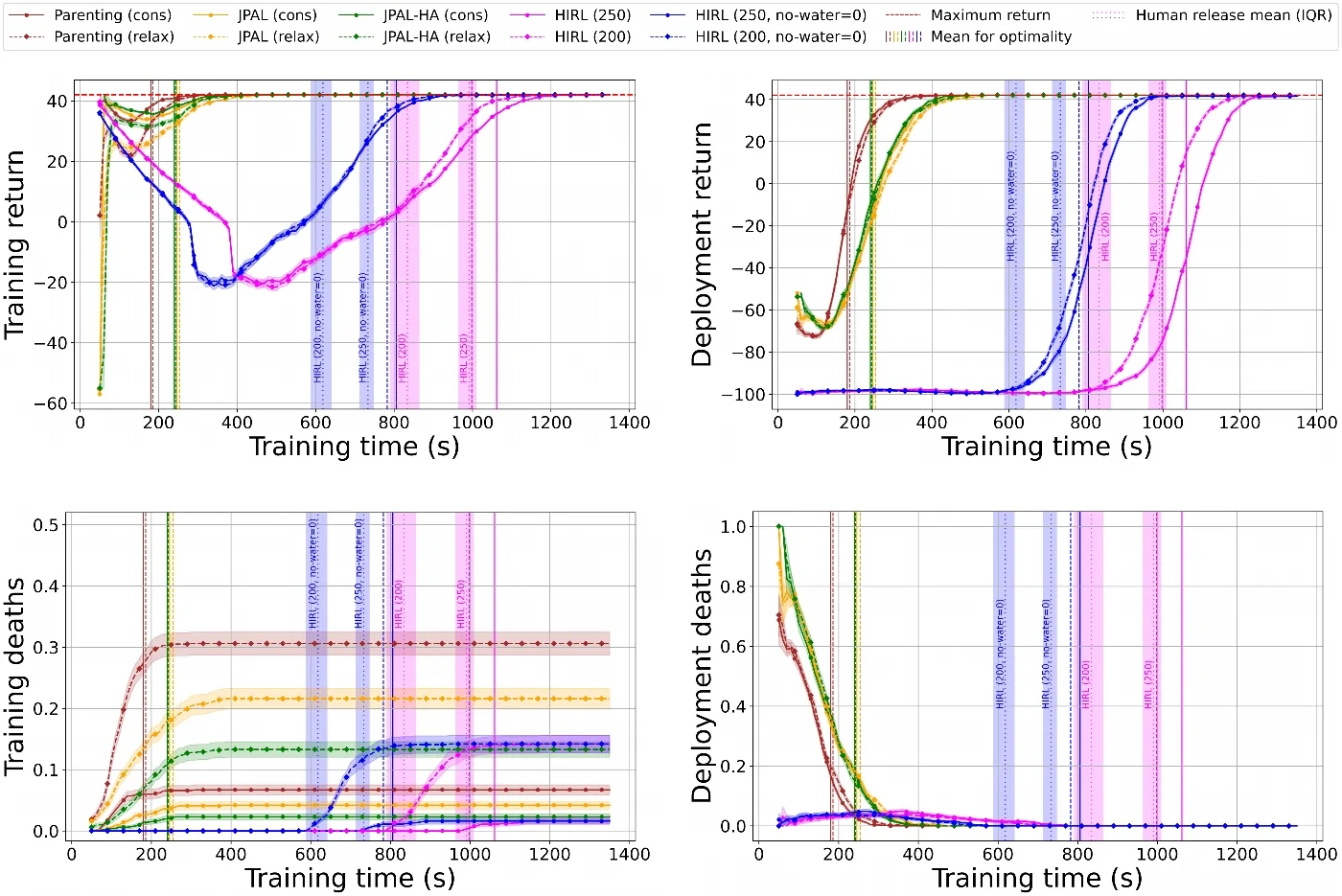
Training/Deployment performance and safety in relation to training time (Configuration of Figure 1)

In addition to the above we also know from our experiments with the real robot (Section 5.8) the time for a robot’s move to a neighbouring cell, which is $t_{{robot}}=2s$. As a result, if we multiply this by the mean number of overall steps until optimality and then add it to the mean human feedback time, we can have the actual training time until optimality for each method. This is important because different algorithms have different numbers of overall steps, throughout which the human still needs to be present, even if they do not provide input at every step. Given all the above, we can now not only compare the mean human input or training times until optimality, but also the behaviour of different metrics during training and compare the algorithms on fixed budgets of human input or training time. This is shown in Figs. 15 and 16 for the original configuration of the Island Navigation (Fig. 1), where for each fixed time budget at 10s intervals, we report, across 1000 trials run until optimality, the average of the training/deployment return or training/deployment deaths of the latest completed episode whose time did not exceed the budget. The shaded region corresponds to *± 1* standard error of the mean (SEM) across trials. The vertical lines correspond to the mean time until optimality for each algorithm, except for the dotted lines in Fig. 16 for the HIRL variants, which indicate the mean time at which the human is released and training continues with traditional RL; the shaded bands around them show the interquartile range (IQR). Solid lines for Parenting, JPAL and JPAL-HA depict the conservative setting (Section 5.1), and dashed lines of the same colour depict their counterparts in the relaxed setting. For HIRL, the solid lines represent the case of releasing the human after 250 user inputs to the catastrophe classifier (enough for nearly 0 training deaths — Fig. 8a), while dashed lines of the same colour represent the same for 200 inputs. The magenta and blue HIRL curves are not different experiments, but simply the blue considers zero human input time at states far away from water.^27^

Appendix D provides more results and discussion. Appendix D.1 focuses on the mechanism for detecting deadly patterns, Appendix D.2 evaluates on more Island Navigation maps and Appendix D.3 on other environments.

Initially, comparing Parenting, JPAL and JPAL-HA, we observe a faster convergence to the optimal return for Parenting than JPAL and JPAL-HA (i.e. the former needs less human input or overall training time). Moreover, we see that if we stopped training at a time budget before the methods’ convergence (e.g. around 150s for human input or 200s for training time) the deployment deaths would be lower for Parenting. However, the opposite happens for the training deaths. Since all methods finally end up to the optimal return and to zero deaths during deployment what matters most is the training deaths.^28^ Also, we can see that the final mean values of the training deaths shown are the same as the ones reported in Table 2. In addition, as we expected from the analysis in Section 5.8, the curves between JPAL and JPAL-HA slightly differ in favour of JPAL-HA, except from the one of training deaths where the difference is notable. In regards to HIRL, we see that even when the input time for feedback in states far away from water is considered zero, if we compare HIRL with a preference-based method that returns approximately the same number of training deaths, it is outweighed prominently in terms of training time. For example, comparing JPAL-HA(cons) and HIRL(250, no-water=0), from Fig. 16 it is evident that the many more steps the latter requires the user to stay in the loop until they can be released make its training much less efficient in terms of overall training time. This was indicated in Section 5.7 and Table 5, but now we can also observe it in actual training time, e.g. by comparing, for the above example the approximately 730s required for HIRL human release with the around 230s required for JPAL-HA optimality.

## Discussion

### Related work

There are a variety of techniques that use a human to help an agent learn a good policy. Most focus on sample efficiency, but some also consider safety.

A standard approach to incorporating human input is learning from human demonstrations, such as imitation learning [31] or inverse reinforcement learning [32–34]. Learning from demonstrations can be highly effective, especially when high-quality demonstrations are available and in large quantities. In recent years the increased availability of large-scale datasets has made this more feasible in several domains, including robotics [35]. Imitation learning can reduce the need for costly or potentially unsafe online exploration by training agents to reproduce behaviours from previously collected demonstrations. In this way, autonomous systems can acquire useful initial behaviours before undergoing further exploration or fine-tuning. Although imitation learning constitutes the foundation in many domains, including large language models (discussed below) and safety-critical ones like autonomous driving, it is often part of a broader pipeline that incorporates at later stages traditional RL or learning from other forms of human feedback, such as human preferences. These later stages can help fine-tune behaviour and improve reliability and alignment with human objectives [36–39]. We note that in this work demonstrations could have more easily solved the tasks considered; however, our focus was to study how the ideas of justifications and hypothetical actions could improve safety in preference-based learning.

Another approach is *reward shaping*, where the human feedback is a numerical reward over an action or trajectory, which can replace or be combined with the environmental reward [27, 40–43] in order to model a reward function. In general, reward shaping does not deal directly with the problem of safety and it is also not always clear if a human can define whether an action or trajectory is good (or how good) or not and return an accurate reward signal. The *ReQueST* (Reward Query Synthesis via Trajectory optimisation) framework, however, uses reward shaping in [27] and also addresses safety through hypothetical behaviours that are generated by a generative model of initial states and a forward model of dynamics trained on off-policy data. These data can be safe human trajectories or past failures. Then the human assigns rewards to the hypothetical behaviours and a neural network is trained to predict them. This is the work from which HAs were inspired for JPAL-HA, with the main difference being that we sample hypothetical actions from the policy model directly in critical situations. The ReQueST framework was scaled up in [17] to complex 3D safety-critical environments using, instead of reward shaping, a more sample-efficient type of feedback, *reward sketching* [44]. In the latter, humans are shown a video sequence of frames and are asked to draw a ‘reward curve’ based on the progress of the task. Although reward sketching is probably the most efficient type of human feedback, it works only with raw pixels and requires a lot of engineering and fine-tuning. Moreover, although ReQueST has been evaluated in complex 3D simulated environments, it has not been tested in the real world, where different challenges may occur, such as the acquisition of adequate off-policy data.

An approach directly dealing with safety is Human Intervention RL (HIRL), where the human continuously supervises the agent and blocks catastrophic actions. HIRL [22, 45] (described in more detail and compared to JPAL-HA in Section 5.7) builds a binary catastrophe classifier via supervised learning, which then takes over from the human trainer. Our work has a similar motivation, but we only seek input from the human trainer when the agent has less certainty about good action choices to make. However, HIRL has been shown to scale poorly to higher-dimensional state-action spaces due to the prohibitively large supervision time needed, deriving from the inherent sample inefficiency of RL (this was evident even for a small environment in Section 5.7). A recent work [46] related to interventions and human demonstrations has tried to combine a number of mechanisms on an unmanned ground vehicle navigation task, by learning in simulation and deploying in the real world. Although it shows improved sample efficiency, this approach has the limitations of the simulation-to-reality gap which makes its use limited.

A very sample-efficient technique, which works in all domains, and has been widely adopted over the last years is learning from *human preferences*. It is essentially an *active learning* [47] method, with the goal to eliminate human feedback time in applicable magnitudes. Instead of picking one greedy action (or trajectory) from a policy model and expect from the human to give a scalar reward to that, it would be more efficient to sample two actions (or trajectories) and ask the human which one they prefer. This approach naturally aligns the agent’s learning with the human objectives and values. For the agent, this feedback helps distinguish which action better matches human goals, while for the human, it is easier and more intuitive to express a preference between two options rather than trying to assign an absolute score, which can be ambiguous^29^. This is also the natural way people communicate: a child having in mind two supermarkets to buy things from, they would not ask their parent: ‘How good is the one I think is the best?’ (as in reward shaping), but they would ask: ‘In which of the two supermarkets should I go?’ (reflecting human preferences). A noticeable work that formalises the framework of human preferences with deep RL is [48], which fits a reward model to the comparisons between segments of sampled trajectories. Specifically, a behavioural policy (trained by traditional deep RL) generates these trajectories and some segments (short sequences of a trajectory) are extracted from them. Then the human compares pairs of segments, giving their preference to the one or to the other. With these data, a deep reward model is trained by supervised learning, where the objective function to be minimised is the cross-entropy loss between the model’s prediction and the human’s preference. The output of that model (scalar reward) is then given to the agent to learn via a traditional deep RL algorithm. The main differences between [48] and our work are:

1. Christiano et al. [48] use segments of trajectories starting from different initial states for a preference query; instead, we use state-action pairs.
2. JPAL-HA (as well as Parenting and JPAL) directly learns a policy model by supervised learning from an increasing dataset of human preferences, omitting the reward model. As discussed in [11] building a reward model is less aligned with alleviating the safe exploration problem, because the maximisation of the reward function with RL can lead to unsafe states if it has not been encoded to the reward with a preliminary mechanism that unsafe states should be avoided. However, direct policy learning using state-action pairs, compared to learning a reward model using segments of trajectories, may limit generalisation, and capturing long-term consequences of actions (Section 6.3).

Although with the technique of learning from human preferences the amount of human feedback is decreased by several orders of magnitude, during RL with the learned reward model, the agent’s interactions with the environment still remain a lot. Recent papers work on the direction of improving sample efficiency both in terms of the human burden and the number of overall interactions, using different intelligent ways, such as the use of human demonstrations for a good initialisation of policy [49], unsupervised pre-training and off-policy RL with relabelling [50], semi-supervised reward learning using a large number of unlabelled samples with data augmentation [51], exploration based on the learned reward’s uncertainty [52], adopting the performance of the Q-function as the learning target at the reward learning [53], pre-training a reward model with trajectories from a family of prior tasks and quickly adapting it online with fewer human queries to new tasks [54], using transformer architectures to model human preferences by capturing non-Markovian reward dependencies across trajectory segments [55], and leveraging optimal transport for transferring preference labels from source tasks to target tasks without extra human queries [56]. Although these works are not oriented towards dealing with safety, in Section 6.3 we discuss future directions that justifications can be incorporated into them.

Preference-based RL has also been remarkably used over the last years in natural language processing (NLP). In text summarisation, [57] collects a large dataset of comparisons between summaries from human contractors, trains a reward model from these comparisons to predict the preferred summary and uses this learned reward function to update by RL a summarization policy. The GPT (Generative Pre-trained Transformer models trained to predict the next token in a document, i.e. when having a dialogue with a user) large language models (LLMs), also use the same method of learning a reward function from human preferences^30^. The first release, InstructGPT [36] is built in three steps: (i) a prompt (i.e. ‘say 3 things you should do before going to bed’) sampled from OpenAI’s prompt dataset; labellers demonstrate the desired model behaviour (i.e. 1) brush your teeth, 2) wind down, 3) limit screen time) and these data are used to fine-tune GPT-3 with supervised learning; (ii) a prompt and some outputs are sampled from the fine-tuned model and labellers rank the outputs from best to worst; these rankings are essentially the human preferences which are used to train the reward model; (iii) a policy is optimised against the reward model using traditional RL algorithms (i.e. PPO [59]). From the first step, known as supervised fine-tuning (SFT) come primarily the model’s capabilities. However, the other two steps, known as RL from human feedback (RLHF) which is essentially the idea originated from [48], are crucial to align the model with the user’s objectives. Later releases of GPT had similar architectures, with some differences in step (i), but more advanced capabilities than a simple dialogue system, such as producing code (ChatGPT [60]), understanding image inputs (GPT-4 [61]), and understanding and generating image and audio content (GPT-4o [62]). The LLaMA series has seen similar advancements, with the latest models [63, 64] building on the foundation of earlier ones [65], by incorporating RLHF to improve alignment with human preferences, ensuring safer and more ethical outputs, particularly in tasks like multilingual dialogue and long-form text summarisation.

All the above systems tackle safety through mechanisms such as guardrails, content filtering, prompt moderation, and safety protocols that detect and mitigate harmful or biased outputs. A more safety-guided method is [66], which introduces Rule-Based Rewards (RBR) to explicitly enforce desired behaviours by incorporating fine-grained, composable rules during RL. This approach reduces reliance on extensive human feedback, offering a more precise and scalable way to align LLMs with safety objectives while minimising over-cautious responses. Closely, the Safe Reinforcement Learning from Human Feedback (Safe RLHF) framework [67] decouples helpfulness and harmlessness during fine-tuning by optimising these conflicting objectives separately. This method employs a Lagrangian approach to dynamically balance safety constraints (harmlessness) and model performance (helpfulness), adapting the trade-off as needed during training. Our paper can contribute in the same direction of the above methods. We allow preferences to be accompanied by a justification. We assume that incorporating justifications alongside the preference rankings of RLHF could further improve alignment and safety, with further examination needed to determine how to best integrate them into the loss function for more efficient learning.

However, RLHF faces challenges with sample efficiency and stability, as gathering high-quality human preference labels, training a reward model and using RL to optimise the policy against this reward model is a costly and complex procedure. To address this, alternatives have been explored recently. In Reinforcement Learning from AI Feedback (RLAIF) [68], feedback is provided by an off-the-shelf LLM instead of human annotators, which generates preference labels based on task-specific prompts. This method has shown comparable performance to RLHF in tasks like summarisation, significantly reducing the reliance on costly human input while still aligning models with human preferences. Direct Preference Optimization (DPO) [69] offers a more stable and sample-efficient method by bypassing the reward model and RL altogether. DPO directly optimises human preferences through a simple classification loss, eliminating the need for complex RL training loops and large-scale sampling from the model. Our approach, which also directly optimises the policy from preferences, although using a different loss and in a different context, has similar advantages in simplicity, efficiency and alignment with avoiding unsafe behaviour. It also has similarities with the regret-based preference model [70, 71]. That approach challenges the assumption that human preferences are based on the sum of rewards within trajectory segments, but instead suggests that preferences are driven by the regret, which measures how much a sequence of actions deviates from the optimal behaviour. The same could be considered for the preferences in our case, where state-action pairs are segments of length 1. Moreover, the regret-based approach [70, 71], similarly to ours and DPO [69], shifts away from optimising a proxy reward function, but instead it allows for direct optimisation by choosing actions that minimise the regret. Finally, a number of more recent works combine the power of LLMs and human feedback in safety-critical tasks, such as in robotics [72–74].

Our work was most similar to Parenting [11] with the main similarities being the query selection method (Section 3) and the direct policy learning from human preferences over state-action pairs (Section 4.3). We optimised Parenting in a couple of ways such as the mechanisms of recording and asking ATFQs, avoiding bothering the human with repeated queries, and different details in the architecture. We differentiated our approach from Parenting through the addition of our main novelties, i.e. the ideas of justifications and hypothetical actions. However, as we discuss in Section 6.3, these ideas are not necessarily tied to Parenting and can be explored in a more general context.

Our research regarding safety concerns has also a similar motivation with the large body of literature in safe RL that does not rely on human feedback [4–9, 75–80]. These works use safety constraints which have to be respected during training and deployment, and are mainly modelled through the framework of constrained Markov decision processes (CMDPs) [16], where an agent maximises the expected cumulative environmental reward while ensuring that an expected cumulative cost is less than a threshold. For example, CPO [5] optimises policies subject to long-term constraints using trust-region updates; Wachi et al. [79] propose MASE, which uses uncertainty quantification and an emergency-stop mechanism to avoid unsafe actions during training; and SHAPO [80] makes on-policy safe RL updates to be pessimistic with respect to the actor’s epistemic uncertainty (makes the policy update more cautious about rare unsafe actions). Other examples include Lyapunov-based constraints [6], barrier-function-based safe control [7, 76], curriculum-based safe exploration [8], local generalised linear approximations for safe policy optimisation [77], and safety-editor policies [78]. Although these methods are highly relevant to safe exploration, they assume, apart from the safety constraints, the availability of an explicitly specified (well-defined) environmental reward that defines the optimisation objective. This is a fundamentally different assumption from our setting, where no handcrafted reward is observed during learning, but all learning comes from the human via preferences and safety justifications before an action is taken.

### Modelling human error

In this work, we assumed that human trainers possess a reasonable understanding of the environment and are capable of providing correct feedback in response to the agent’s queries. This assumption is particularly appropriate for controlled environments like the Island Navigation, where humans can consistently recognise unsafe states and provide accurate feedback. However, as tasks grow more complex, such as helicopter flight or intricate robotics tasks, the potential for human error increases, and the performance of the learning process may degrade accordingly.

In [48], the authors used a 10% probability of random mistakes in preference answers. This approach highlights the impact of uniform error rates and demonstrates that agents may still converge to a near-optimal policy despite noisy feedback. A more sophisticated approach to modelling approximately-rational human feedback uses the Boltzmann distribution, where the probability of selecting an option is linked to its exponentiated expected reward, tempered by a rationality parameter *β*. In [81], human feedback is modelled as a *Boltzmann-rational policy*, where the probability of selecting a particular action $c^*$ from a set of options *C* is proportional to $\exp ( \beta \cdot r(\psi (c^*)))$. Here, *ψ* is a *grounding function* that maps human choices to trajectories, and $r(\psi (c^*))$ represents the expected reward for a given choice. This formulation provides a nuanced understanding of how humans act in a way that approximately maximises rewards, while accounting for bounded rationality and noise. The Boltzmann policy distribution [82] extends this by addressing *systematic suboptimality*, where human feedback reflects consistent biases rather than randomness. Trainers may favour suboptimal trajectories due to habits or limited understanding, and adjusting the rationality parameter *β* models this bias more accurately by coupling actions over time rather than treating them as isolated decisions.

With regard to preference learning, [83] employs a Boltzmann-like model to simulate noisy preferences. Similar to the Boltzmann distribution, this approach uses *β* to model the rationality of synthetic humans. Akin to the uniform noise seen in [48] it also uses an *ε* as an error rate to capture the likelihood of accidental mistakes. The study shows that methods such as PEBBLE [50] noticeably degrade performance when wrong labels are provided, while the extent of the degradation varies based on the specific design choices and task setups. In our method, where preferences directly shape the policy without the use of a reward model, noisy feedback could have a more immediate impact, possibly increasing the number of steps required for learning when non-safety-critical errors occur. Moreover, if a critical safety error occurs, such as falsely neglecting to provide a warning, then the safety of the agent can be compromised, as it may immediately execute a catastrophic action. However, the use of justifications and hypothetical actions may reduce the likelihood of human errors (at least accidental ones), as they explicitly ask the user to reflect on whether an action is unsafe and thus act as an additional layer of cognitive reasoning after the initial preference.

In [84] several techniques are introduced to handle noisy and inconsistent preferences, including latent space regularisation and confidence-based reward model ensembling. Although these ideas use a reward model, they could be possibly adapted for our case, where we learn a policy directly from preferences. For instance, rather than using a reward model for ensembling based on confidence, we can adapt this idea to weigh human feedback based on the reliability of each specific feedback instance. That is, if a particular preference and justification combined answer aligns well with the general trend or shows consistency across similar situations, it would be assigned a higher confidence score, whereas noisy or contradictory answers would be down-weighted. This ensures that even if some feedback is unreliable, it will not disproportionately affect the policy learning process. Taking it a step further, unanswered queries or queries with high uncertainty responses could be batched and reissued to the human at a later time, when their understanding of the environment would have improved. Additionally, if dealing with feedback from multiple annotators, each annotator’s input could be adjusted according to their demonstrated rationality or expertise level, giving more weight to answers from more reliable sources.

### Justifications and hypothetical actions beyond gridworld environments

We saw that the inclusion in preferences of justifications and HAs can significantly improve safety (and possibly performance in general if used not only in terms of safety – Appendix B). In this section, we discuss whether and how these ideas can work beyond discrete low-dimensional environments like the Island Navigation and provide directions after JPAL-HA. In JPAL-HA we integrated the ideas of justifications and HAs with a couple of core elements from Parenting. These were (i) the online querying method ($(p_{{BTFQ}})^{f(s_{t})}$ in Section 3); (ii) the direct policy optimisation (Equation 2); and (iii) the issuing of queries over state-action pairs (instead of over trajectories). As a result, in order to understand whether justifications and HAs can work beyond gridworld environments we need to ask first whether the additional time and human burden are significant from the justifications and hypothetical queries in themselves; and second whether these techniques can be integrated in other scalable settings regarding (i)-(iii), i.e. to work (i) offline, (ii) with a learned reward model, and (iii) with queries over trajectories.

Regarding the first question, we have indeed seen that adding justifications only slightly affects human burden and time as it requires a single additional bit added to a preference query. In Section 5.8, we found that in actual time it took the participants around 30% longer to complete training when justifications were added. In other words, any algorithm which can scale in time with plain preferences will not face an issue by adding justifications; in the worst case the time will double but still scale. As for HAs, scaling can be affected for large actions spaces, but only if we intend to investigate a very big portion of actions from an identified dangerous state. The formula in Equation 1 provides a flexible way to moderate this, by customising $\alpha _{{safe}}$, *k* and $\alpha _{{stop}}$ to the action space size and the safety severity of the environment. Thus, justifications and HAs in and of themselves do not limit scalability.

Regarding the second question, although the settings (i)-(iii) we used in JPAL-HA were convenient for the gridworld, they may often restrict applicability. For example, as for (i), online querying and pausing to answer can be both sample-inefficient and not even possible in real-time safety-critical domains like autonomous driving. Relating to (ii), Equation 2 risks generalisation, as preferences may not transfer across similar states or tasks. That is, distribution shift becomes an issue for JPAL-HA, and normally additional human feedback would be needed if the testing distribution differs from the training one (e.g. the configuration of the Island Navigation environment changes). Instead, using a reward model provides a latent global representation that can often better generalise human feedback and enable the development of a more transferable policy. Finally, regarding (iii), using a reward model over trajectories captures the long-term consequences of actions by considering how actions in one state affect future states and rewards, providing more strategic understanding of the task. Taking into account these factors, we now present three directions that justifications and HAs can be used more broadly without the aforementioned restrictions of JPAL-HA.

First, although it is not the main focus of this paper, we consider the case where the environment is not safety-critical, and justifications, as explained in Section 4.1 and Appendix B, are not necessarily related to safety. In such cases, where experience from the agent can be safely gathered directly in the real world, justifications can be easily integrated with preferences when using a method such as PEBBLE [50] or any other work which improved on it [51–56]. Precisely, after some optional unsupervised pretraining stage, experience is stored in a replay buffer. Then segments of trajectories are formed from that experience and shown to the user to provide feedback offline, and build from that a reward model with the Bradley-Terry model. As justifications only impact the preference ground-truth label *μ* and not the preference predictor (i.e. Equation 3 in [50]), it is simple to integrate them with preferences, just by using the mapping $P \times J_{P} \rightarrow \mu$ (Section 4.1) or by using the generalised formula for *μ* for an arbitrary number of justifications (Appendix B). With this integration, performance and alignment are expected to improve. The framework of HAs, since the environment is not safety-critical and the queries are not answered online, is not anticipated to be as useful or applicable; however, the idea of focusing on a region where an important justification was raised with some other mechanism could still be considered.

Second, returning to the case of safety-critical environments, the restriction related to (i) online querying and pausing to answer could be waived, if we assumed the resource of a fallback safety mechanism. For instance, during autonomous driving, in high-risk situations where the agent cannot query the human trainer immediately, it could rely on a temporary guardrail, such as slowing down or halting, while at the same time batching the queries it was planning to ask. The ‘parenting’ querying method of issuing a query with a higher probability in less explored states (Section 3, and more formalised in Appendix A) could still be applied, but the queries would be batched for slightly later. During following less critical moments, the agent could send the batched queries to the human along with a visual snapshot or simplified map to recall the earlier situation (this snapshot could be shown for example on a display in the car). The human would then be able to respond with preferences and justifications, and also use the HAs framework (i.e. for a given warning over a preference, a follow-up justification query would be ‘if you had turned left at that intersection a few seconds ago, would that have been safe’). This offline querying would allow the agent to refine its policy without interrupting the real-time flow, gradually replacing the broad fallback rules with more tailored, safe actions learned through human preferences and justifications. However, potential issues with generalisation due to (ii) direct policy optimisation and (iii) querying over state-action pairs would still remain.

Third, again for safety-critical environments, we may assume the resource of a dynamics model of the environment or the ability to learn one from safe human trajectories, past failures or controlled unsafe trajectories (i.e. a vehicle safely colliding with soft obstacles or a drone landing on padded barriers) as in [85] (*world model*). Then inside this world model (not in the real world), a reward model could be safely trained from human feedback, i.e. by using trajectories drawn from the dynamics model. This reward model would then be used to either learn a policy in the world model with traditional RL (i.e. as in PEBBLE) and transfer it to the real world; or deploy the agent directly in the real world with *model predictive control* [86], by using the dynamics model to sample some trajectories and the reward model to pick the one with the highest predicted return, as done in [27] and [17]. The human feedback for building the reward model could include the justifications along with the preferences, where a ‘warning’ would be assigned when at least in one of the two trajectory segments the agent had passed from unsafe states, and a ‘no warning’ would be given if both trajectories had been safe. In that case, *μ* would be shaped differently again (than with plain preferences), and thus, safety would be better encoded in the learned reward function. Although, similarly to the first case, justifications can work with (i) a human answering completely offline, (ii) a learned reward model and (iii) queries over trajectory segments, the limitation in this case is the acquisition of data to construct an accurate dynamics model and the reality-world model gap, since the reward model is learnt not in the real, but in the simulated world. The HAs framework (hypothetical trajectories in this case) although not as straightforward as in the second case, could possibly be used again with certain modifications, if the issued preference and justification queries referred to trajectory segments starting from the same state. This would be easier if we had both the resources of a fallback mechanism and a dynamics model, so that the user could reflect on a slightly earlier dangerous moment and respond to justifications over *hypothetical trajectories* generated from the dynamics model and the current policy.^31^ To conclude, although we introduced justifications and HAs in a simple gridworld case, we believe they can be integrated into more complex scenarios, the evaluation of which is left for future work.

## Conclusion

In the context of research which uses human preferences to train an agent safely, we proposed two novel ideas: the use of justifications and hypothetical actions, which, combined into JPAL-HA, based on our evaluation improve safety significantly and consider human burden. We focused on the safe exploration problem and demonstrated through experiments in a simulated environment that both the use of justifications and hypothetical actions significantly improves safety during the learning process. We showed that it does not affect the human burden under the assumption that justifications and hypothetical action queries place a marginal additional cost. In cases where we do not make this assumption, such as in the user experiments with a mobile robot, the use of hypothetical actions still slightly reduces the overall training time and ensures it remains within acceptable limits.

## Figures and Tables

**Fig. 17:**
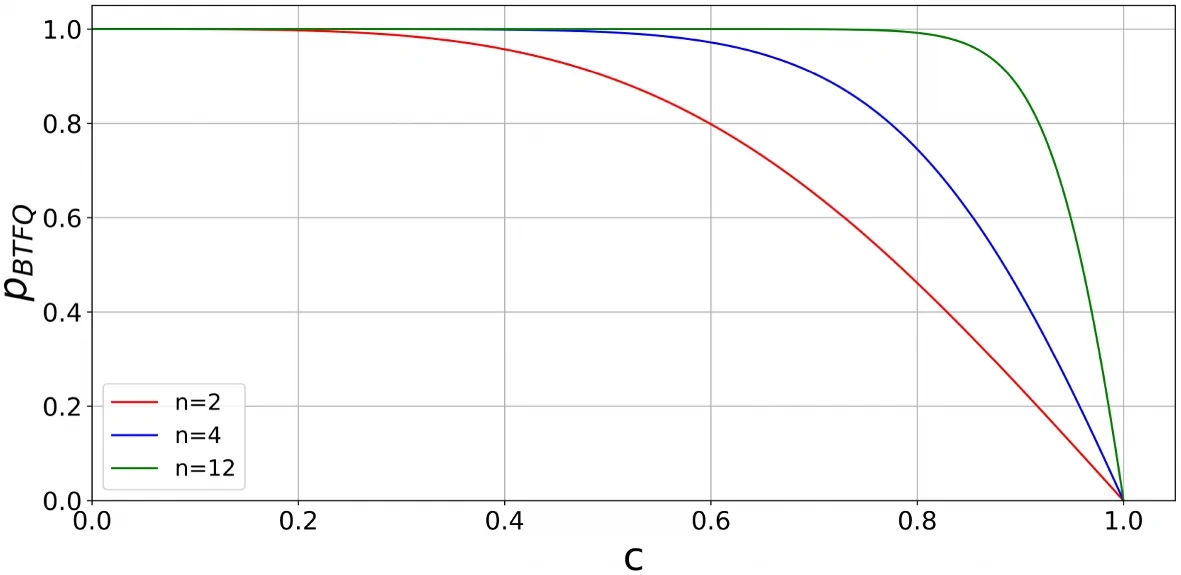
$p_{{BTFQ}}$ (probability of asking a BTFQ) vs *c* (confidence) at a specific state or region

**Fig. 18:**
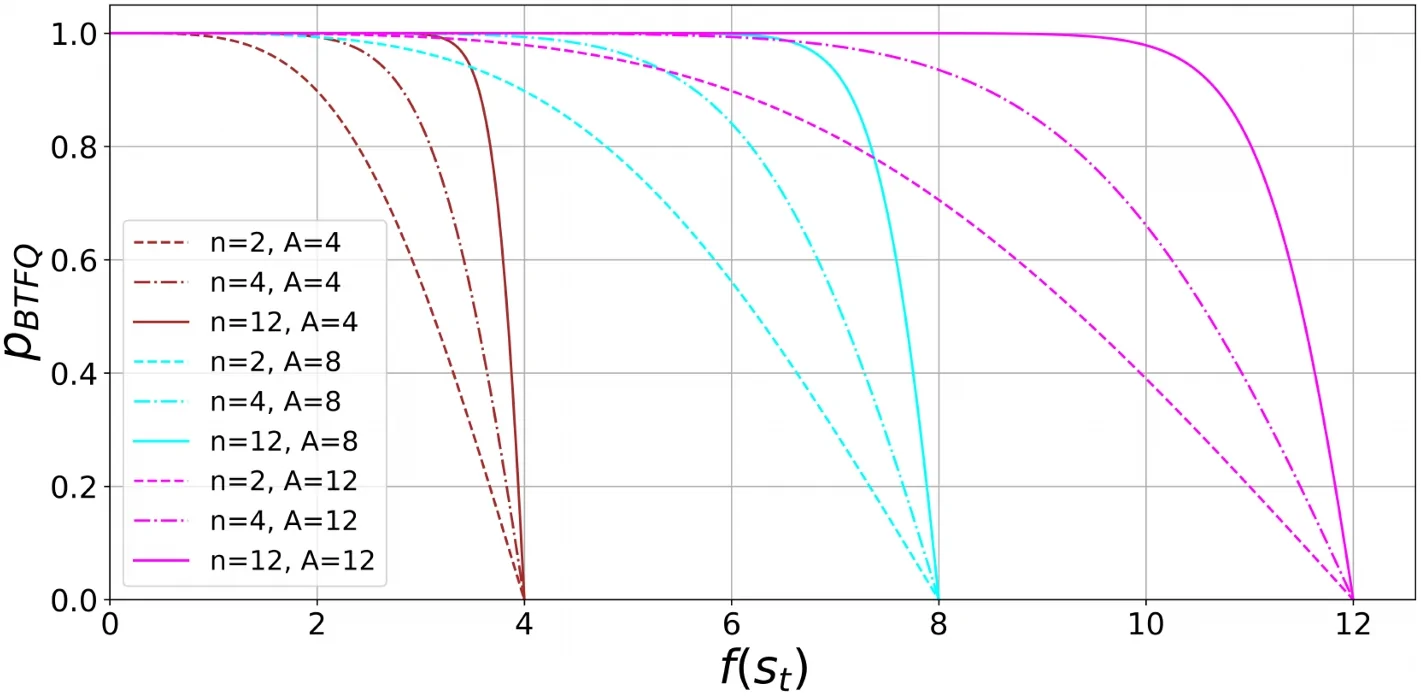
$p_{{BTFQ}}$ (probability of asking a BTFQ from a specific state) vs $f(s_t)$ (number of actions that have been explored from $s_t$)

**Fig. 19:**
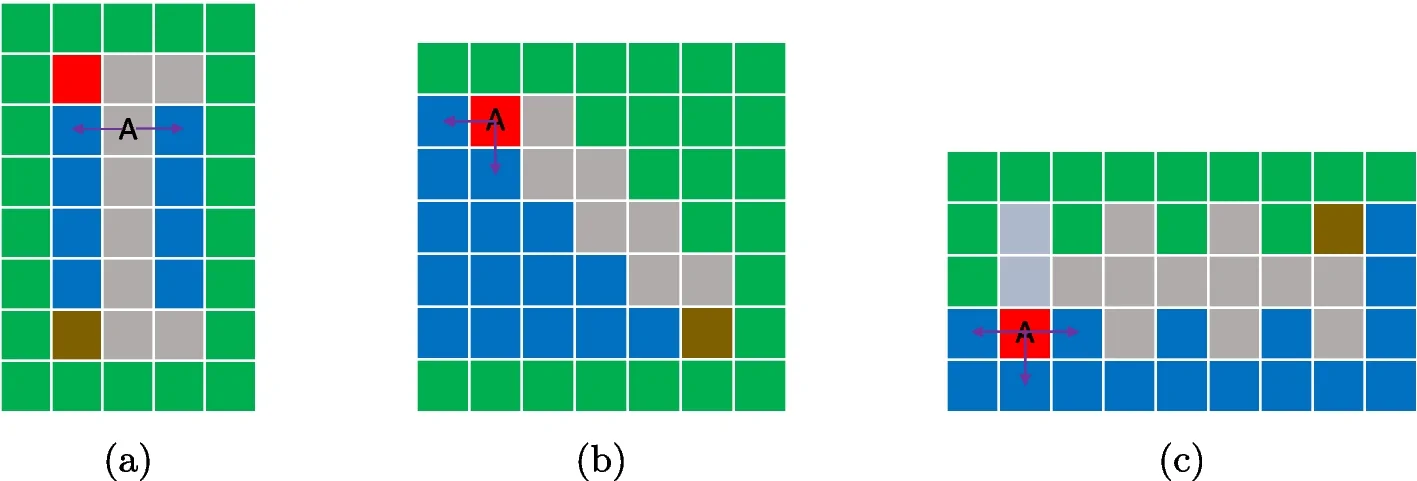
Characteristic examples of detecting deadly patterns

**Fig. 20:**
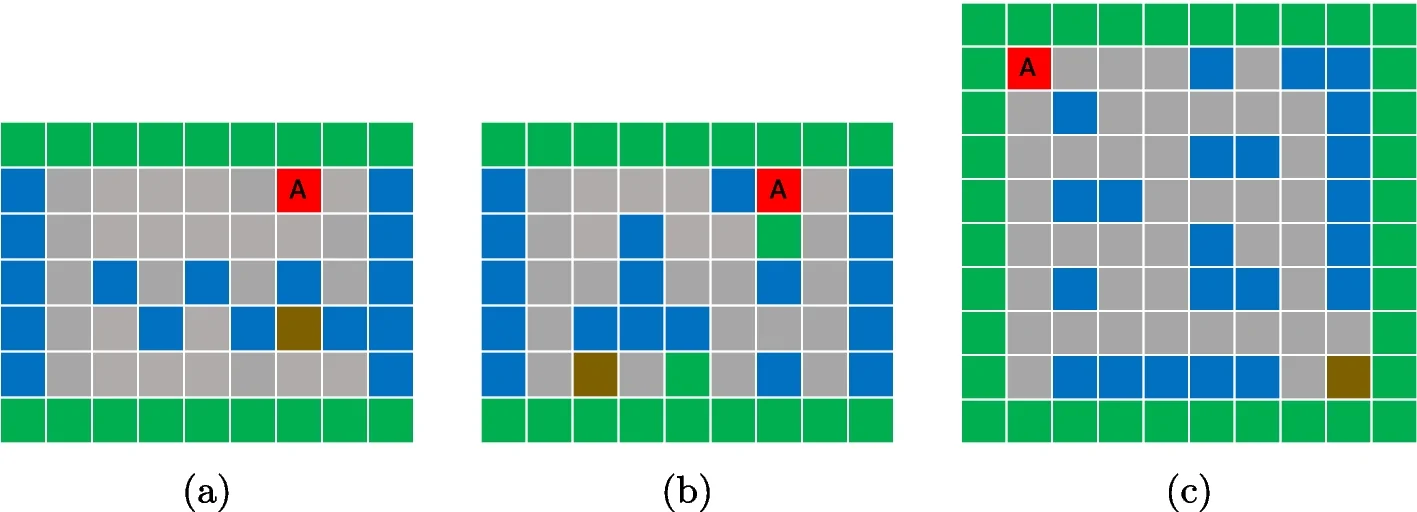
More Island Navigation maps

**Fig. 21:**
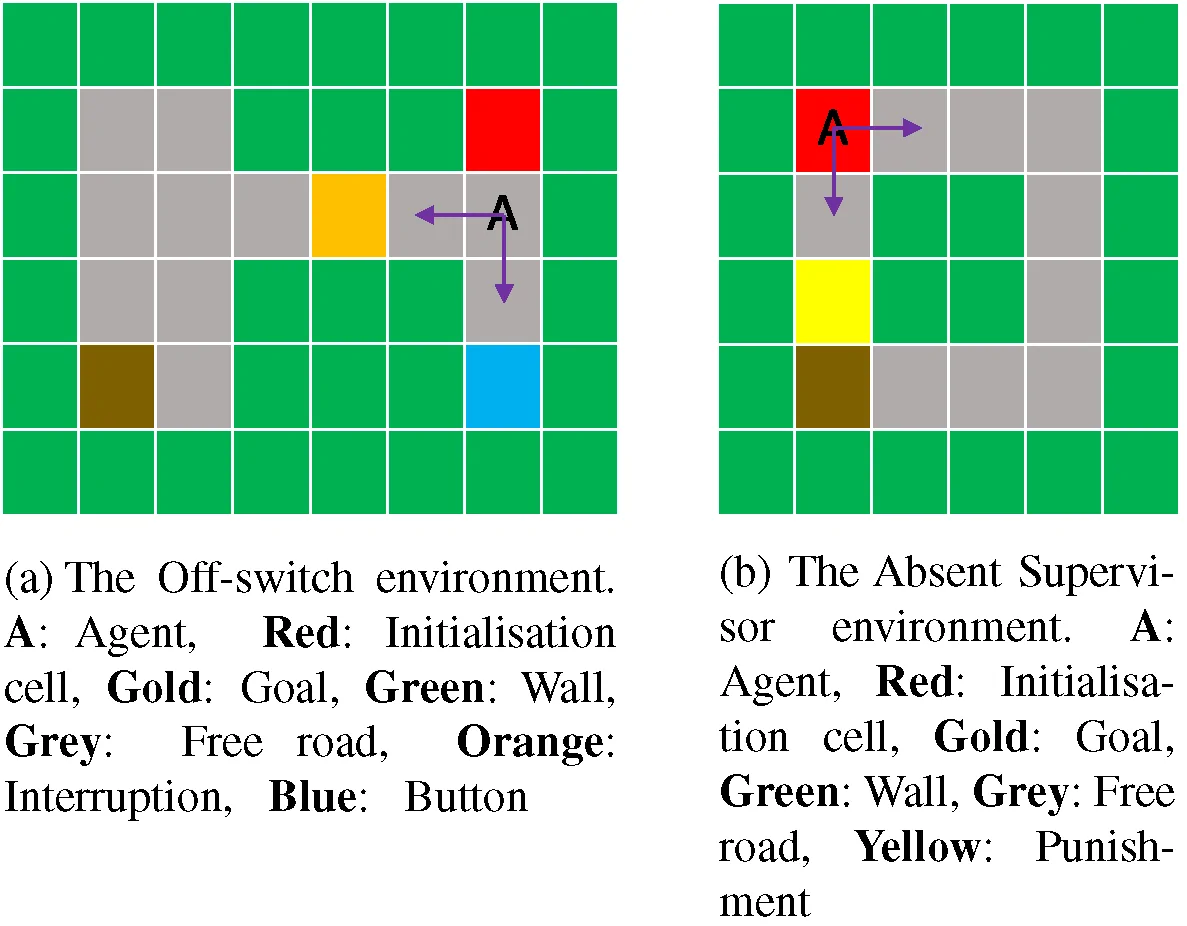
More environments in which JPAL-HA was evaluated

## Data Availability

All data supporting this study are openly available from the University of Southampton repository at https://doi.org/10.5258/SOTON/D2496

## Appendix A Generalising the querying method to large and continuous spaces

In Section 3, we decided to use the same idea as [11] regarding the decision to issue a BTFQ (i.e. issue a BTFQ with probability $(p_{{BTFQ}})^{f(s_{t})}$ with $p_{{BTFQ}}$ being a hyperparameter close to 1), which was convenient for the evaluation in a discrete and low-dimensional space, such as that of the Island Navigation gridworld. In this section, we present a more generalised formula based on confidence that can be applied to larger and continuous spaces.

In this formulation, to manage the balance between human oversight and agent autonomy, we introduce a query probability formula based on *confidence**c ∈ [0, 1]*. The confidence term *c* reflects how familiar the agent is with a state or region, and directly influences the probability $p_{{BTFQ}}(s_t)$ that the agent should issue a BTFQ from state $s_t$ to the human^32^. The relationship of $p_{{BTFQ}}(s_t)$ and *c* is modelled in a way that mirrors the shape of a Butterworth filter response up to the cutoff point. Here, the y-axis represents $p_{{BTFQ}}$, and the x-axis represents *c*. The relationship should smoothly decrease from $p_{{BTFQ}}=1$ at *c = 0* to $p_{{BTFQ}}=0$ at *c = 1*. To achieve this, we modified the Butterworth filter by scaling its response. Specifically, from the original Butterworth magnitude function $\frac{1}{\sqrt{1 + c^{2n}}}$ we subtracted $\frac{1}{\sqrt{2}}$ to shift the value at *c = 1* (originally $\frac{1}{\sqrt{2}}$) to 0. We then divided it all by $1 - \frac{1}{\sqrt{2}}$ to rescale the entire response so that it still remains 1 at *c = 0*, maintaining a smooth and controlled decline (Fig. 17). The resulting relationship after simplifications is given by the formula:

3 $$\begin{aligned} p_{{BTFQ}}(s_t) = \frac{\sqrt{2}}{\sqrt{2} - 1} \left( \frac{1}{\sqrt{1 + c^{2n}}} - \frac{1}{\sqrt{2}} \right) \end{aligned}$$

Here, *n* is a tuneable parameter (analogous to the order of the filter) that governs how aggressively the query probability decays as confidence increases, and *c* is a measure of the agent’s familiarity with the environment at the specific state or region. This confidence can take various forms, depending on the environment and the task.

The parameter *n* allows us to control how much time and effort we want to devote to human queries (reflects the willingness to answer queries). Higher values of *n* will slow the decay of the query probability, meaning the agent will continue to ask queries for a longer period, even as its confidence increases. This is particularly useful in high-risk environments, where safety is a primary concern, and the cost of an incorrect action could be catastrophic. On the other hand, lower values of *n* will result in a faster decay of the query probability, allowing the agent to query less frequently as it becomes more confident. This is suitable for lower-risk environments where autonomy is preferred and the human burden should be minimised.

In the discrete case, the action space size *A* is introduced as a decisive factor, especially when it is large. The familiarity of a state $f(s_t)$ can be considered the number of actions that have been explored from $s_t$. Then, we can define the confidence as:

4 $$\begin{aligned} c = \frac{f(s_t)}{A} \end{aligned}$$

This formulation reflects the agent’s increasing confidence as it explores more actions from a state. When the number of explored actions *f* equals the total action space *A*, the agent reaches full confidence, meaning the query probability becomes zero (Fig. 18). Although this approach provides a straightforward way to manage the exploration of action spaces in discrete environments, more advanced and refined ways could be explored, such as incorporating in confidence *c* the certainty from the agent’s policy.

In continuous spaces, a possible way to extend the concept of state familiarity is by utilising a fixed-radius nearest-neighbours approach. Instead of counting queries or actions explored from a specific state as in the discrete case, we count the number of previously visited states within a region of radius *r* around the current state. In other words, for each state $s_t$ visited by the agent, we define familiarity as the number of neighbours (previously visited states) that lie within a distance *r* from $s_t$. Mathematically, this is expressed as:  where $d(s_t, s_i)$ is the distance between the current state $s_t$ and a previously visited state $s_i$, *N* is the number of visited states, and  is the indicator function, which equals 1 if $d(s_t, s_i) \le r$ and 0 otherwise. The number of neighbours is then normalised by a predefined value $N_{\text {required}}$, which represents the number of neighbours required for the agent to feel fully confident in the region defined by radius *r*.

The confidence *c* is then calculated as:

6 $$\begin{aligned} c = \frac{\text {Neighbours}(s_t, r)}{N_{\text {required}}}, \end{aligned}$$

and is capped at 1, meaning that when the number of neighbours reaches $N_{\text {required}}$, the agent is fully confident in the region. When the number of neighbours is low, the confidence *c* is low, and the query probability $p_{{BTFQ}}(s_t)$ remains high, prompting the agent to query the human more frequently in unexplored regions. As the agent encounters more neighbours within the radius, confidence increases, and the query probability decreases, reducing the human’s burden in familiar regions. Although this method provides a simple and interpretable way to adapt the query probability mechanism to continuous environments, more sophisticated methods such as kernel density estimation [19] could further refine the confidence estimation, particularly in highly dynamic environments.

## Appendix B Generalisation to multiple justifications

This section presents how the idea of justifications can be generalised to an arbitrary number of justifications of different severity. Our goal is to determine the preference label *μ*, i.e. how much the first action is preferred to the second, using more than one justification. In Section 4.1 the only justification was related to safety, and specifically was *warnings* whether the agent would end up in water. In that case, we simplified the implementation defining *μ* in {0, 0.25, 0.5, 0.75, 1}.

As a motivating example, another justification could be related to the level of battery of the robot, and whether it should go to recharge or not. In other words, in a case of low battery of the robot, and a preference query between one action (or trajectory segment) that goes towards a charging station and one that goes immediately towards the goal state, the choice of the first has to be *justified*. We can combine different justifications in one formula, by using the indicator function and prioritising them by importance (severity). For example, the justification regarding safety (actions leading to water), would be considered more severe than the justification regarding battery. When a justification is present, then the justifications of lower severity should not affect *μ*. For example, if the agent queries between an action that leads to water, and one that is safe, it is irrelevant whether the battery level is low. Similarly, if the battery level is low, and one action points towards the charging station, whereas the other points elsewhere, the proximity to the final goal state is irrelevant. As a result, we need some weights to define the severity of each justification, which would generally be standard values for common tasks, though still fine-tuneable by human operators before training to accommodate the peculiarities of the environment.

The goal proximity (i.e. choosing an action because it takes the agent closer to the goal) is effectively also a justification, which we are implicitly considering and need to give a weight too. As a result, for the justifications of safety, battery and goal proximity, we can write:  where $J_s, J_b \in \{0,1\}$ are the justifications regarding safety and battery, respectively, $P \in \{{1^{\text {st}}}, {equal}, {2}^{\text {nd}}\}$ is the preference, and $w_s, w_b, w_g \in (0.5, 1]$ are the justification weights corresponding to the severity of safety, battery, and goal proximity, respectively, with $w_s \ge w_b \ge w_g$. A sensible choice could be $w_s=1$ (meaning it is imperative to prefer actions that do not point to water), $w_b=0.9$ (meaning it is serious to prefer actions that will not leave the robot without battery), and $w_g=0.7$ (meaning it is important to prefer actions that move towards the goal state).

The $J_s$, $J_b$ and *P* receive values from the human input. The user could either be asked about all justifications separately, starting from the most severe one, and answering with a binary signal (similar to ‘Warning’ and ‘No warning’ used in Section 4.1). However, a more efficient approach is to always give the preference, and then give the specific justification (reason) for that preference^33^. For example, on the question ‘*Up or Left?*’ the answer would be: ‘*I prefer****Left****because of****low battery***’; this requires only two ‘clicks’ (one for the preference and one for the justification) instead of a click for every justification. In this case, all the justifications of higher severity would be satisfied (i.e. there are no safety issues; neither *Up* nor *Left* point to water) and all the justifications of lower severity would be irrelevant (i.e. it does not matter if *Up* or *Left* goes faster to the goal as long as the battery is low). Investigating this in more detail:

- If $J_s = 1$, the preference label *μ* is determined by *safety* considerations, with weights: $w_s$ for $P = {1^{\text {st}}}$; $1 - w_s$ for $P = {2}^{\text {nd}}$; and 0.5 for $P = {equal}$.
- If $J_s = 0$ and $J_b = 1$, the preference label *μ* is determined by *battery* considerations, with weights: $w_b$ for $P = {1^{\text {st}}}$; $1 - w_b$ for $P = {2}^{\text {nd}}$; and 0.5 for $P = {equal}$.
- If $J_s = 0$ and $J_b = 0$, the preference label *μ* is determined by *goal proximity* considerations, with weights: $w_g$ for $P = {1^{\text {st}}}$; $1 - w_g$ for $P = {2}^{\text {nd}}$; and 0.5 for $P = {equal}$.

We mention that in the Island Navigation case of Section 4.1, we considered only the justifications of *safety* and the default one of *goal proximity*, with $w_s=1$ and $w_{def}=w_g=0.75$.

The formula can be easily generalised for an arbitrary number *N* of justifications. Let:

- $J_i$ be the *i*-th justification, where *i* ranges from 1 to *N*.
- $w_i$ be the weight associated with the *i*-th justification.
- be the indicator function.
- $J_N$ be the default justification with  being implicitly always 1 without need for human input.
- *P* be the preference.

Then, with decreasing safety severity ($w_1 \ge w_2 \ge \dots \ge w_N$), the preference label *μ* is given by:

### The impact of justifications on safety

We also provide a theoretical explanation of why *policy models learned from preferences augmented with safety justifications can promote safer behaviour than those learned from plain preferences alone*. We show this in the case of a single safety justification (e.g. an action leading to water without other safety concerns), while the extension to multiple justifications follows analogously.

Let $\mu \in \{1 - w_s, 1 - w_{def}, 0.5, w_{def}, w_s\} \subset [0,1]$ be the preference label with:

- $w_s$: the weight corresponding to preferences that are justified from a safety factor (e.g. falling into water).
- $w_{def}$: the weight corresponding to a default justification (e.g. reaching the goal faster).
- $$w_s> w_{def}$$.

Let the embraced memory $\mathcal {X}$ with samples of the state, the two queried actions and the preference label $(s, a^{(0)}, a^{(1)}, \mu )$ be partitioned into:

- $\mathcal {X}_{{safety}}$: samples of queries with at least one unsafe action (justified by a safety factor).
- $\mathcal {X}_{{def}}$: samples of all other queries with both actions safe (justified by the default factor).

Then, following Equation 2, the cross-entropy loss used to train the policy model becomes:

9 $$\begin{aligned} \begin{aligned} \mathcal {L}_{\pi } =&- \sum _{ (s, a^{(0)}, a^{(1)}, \mu _{{s}}) \in \mathcal {X}_{{safety}}} \mu _{s} \log p[P={1^{\text {st}}}] \!+\! (1 \!-\! \mu _{s}) \log p[P={2}^{\text {nd}}] \\&- \sum _{ (s, a^{(0)}, a^{(1)}, \mu _{{def}}) \in \mathcal {X}_{{def}}} \mu _{{def}} \log p[P={1^{\text {st}}}] \!+\! (1 \!-\! \mu _{{def}}) \log p[P={2}^{\text {nd}}], \end{aligned} \end{aligned}$$

where $\mu _{s}\in \{1 - w_s, 0.5, w_s\}$ and $\mu _{{def}}\in \{1 - w_{{def}}, 0.5, w_{{def}}\}$. For non-indifferent safety-justified preferences (i.e. when $P\ne {equal}$ and $J_s=1$), $\mu _s$ is further from 0.5 than $\mu _{{def}}$, since $w_s>w_{{def}}>0.5$. For the binary cross-entropy term, the gradient with respect to the preference logit is $p[P={1^{\text {st}}}]-\mu$. Hence, when the policy model is uncertain, safety-justified samples introduce a higher gradient magnitude, and thus the loss puts more pressure on the policy model to fit the safety-relevant preferences (acting similarly to an importance-weighted objective). As a result, safety justifications encourage the policy model to prioritise safe behaviour, which can explain the fewer unsafe events we observed in the experiments when justifications were used.

## Appendix C Experimental details

### C.1 Parenting, JPAL and JPAL-HA

As described in Section 5.1, Parenting’s components (architecture, learning rate, learning steps per epoch, and the methods for recording and querying ATFQs) were initially fine-tuned extensively and applied to JPAL and JPAL-HA as well to ensure fair comparisons amongst all methods. The policy model $\pi (a\!\mid \! s)$ is the one used in [11]. It is the average of two components. The first is a global component which is a CNN, mapping the board state to the actions; the board state is converted to an one-hot encoding form where, for each object (Agent, Goal, Wall, Free road, Water), a different matrix of zeros and ones is created. The CNN consists of four convolutional layers with 16, 32, 64, and 64 kernels, respectively. The kernel size is *3× 3* with a stride of 1. Padding is used to bring the spatial dimensions to *2× 2* before the final convolution. Each convolutional layer is followed by a rectifier nonlinearity activation. The final hidden layer is fully-connected with 64 rectifier units, and the output layer has 4 neurons corresponding to the number of actions. The second component is a local FNN with input being the type of object in the four neighbouring cells of the agent, one hidden layer of 64 units, and four output neurons.

We tested the Parenting, JPAL and JPAL-HA algorithms with two different hyperparameter settings: a *conservative setting* ($p_{{BTFQ}}=0.95$, $p_{{REC}}=0.8$, $p_{{ATFQ}}=0.8$) and a *relaxed setting* ($p_{{BTFQ}}=0.8$, $p_{{REC}}=0.5$, $p_{{ATFQ}}=0.5$). The key parameter is $p_{{BTFQ}}$, which balances safety with human burden. A higher $p_{{BTFQ}}$ increases the number of BTFQs, enhancing safety but requiring more human input, while a lower $p_{{BTFQ}}$ reduces BTFQs, but potentially increases the risk.

For JPAL-HA, the hyperparameters used for the stopping condition (Equation 1) were $\alpha _{{safe}}=0.5$, *k=2* and $\alpha _{{stop}}=1$. For optimisation, we used the Adam optimiser with a learning rate of 0.001 and the default initialisation of parameters. As the number of samples in the embraced memory dataset $\mathcal {X}$ may grow in each interaction step, we created a rule for the number of training epochs per step, instead of keeping them constant. Specifically, in steps that $\mathcal {X}$ was not extended we only trained for 1 epoch, while in other cases we followed the rule:

10 $$\begin{aligned} {epochs} = \lceil w_{{epoch}} \cdot (\mid \!\!\mathcal {X} \!\!\mid _{ current\_step } - \mid \!\!\mathcal {X} \!\!\mid _{ previous\_step }) \rceil , \end{aligned}$$

where $w_{{epoch}}$ was set to 9 after some experimentation. This helped the algorithm to converge faster to the optimal policy. We also found it slightly beneficial to use a learning rate scheduler, whereby when the embraced memory $\mathcal {X}$ is not updated for 20 steps, the learning rate is increased by 10 and the number of epochs is increased to 40 for that step. In general, we observed that small changes in the hyperparameters $p_{{REC}}$, $p_{{ATFQ}}$, $w_{{epoch}}$ and the learning rate scheduler negligibly affected the output.

To avoid rare trials in which the agent gets stuck in a loop of bumping into the same wall cell or going back and forth between two cells, we implemented a simple mechanism that sensed such occasions, i.e. staying at the same state for more than one step or returning to the previous state. This mechanism during a BTFQ kept the human preferred action and also replaced the un-preferred sampled action with a new action from the agent’s policy model. We did this in a loop until a new entry appeared in the embraced memory $\mathcal {X}$, allowing the training to smoothly continue. We believe this is a sensible and practical assumption that could also be implemented for stochastic environments, i.e. by observing the local area, as the agent could utilise simple memory rules or the human could take action as a special case.

### C.2 Human intervention RL

As described in Section 5.7, Human Intervention RL (HIRL) [22] follows a three-stage learning process: (i) the agent initially develops a policy through standard RL methods while a human supervisor monitors its actions, blocking any catastrophic ones and suggesting an alternative action (typically the best one when they understand the environment); (ii) the agent pauses and a binary catastrophe classifier is trained using supervised learning on the intervention data collected from Stage (i); and (iii) the agent resumes learning, but now the human supervisor is replaced by the catastrophe classifier, which acts as a safety monitor. As the authors in [22] note, in Stage (i) the agent slows down to leave enough time for the human to act. The human’s judgement of actions in each state as ‘safe’ or ‘catastrophic’ creates a dataset used to learn the governor in Stage (ii). During learning in Stage (i) the catastrophic action is also penalised with a high negative reward (-50 in our experiments) as if the action had been really taken so that the agent both learns a policy to avoid catastrophic actions and builds the governor.

For the comparison with JPAL-HA, we used the same technique as the HIRL authors of passing the input state of the classifier through several convolutional layers to output a vector, and then concatenating that vector with the one-hot-encoding vector of the corresponding input action. After the concatenation, three more linear layers were used. We used the sigmoid function at the output, binary cross-entropy loss as the objective function, 200 epochs, the Adam optimiser with learning rate=0.01 and Q-learning as the RL algorithm with the same settings as in Section 5.2. The optimal human behaviour for HIRL was simulated in the same way as for JPAL-HA.

## Appendix D Additional results and discussion

Here, we present additional results and discussion, focusing on the mechanism of detecting deadly patterns (Section 11.1) and further testing in more maps of Island Navigation (Section 11.2) and other environments (Section 11.3).

### D.1 Detecting deadly patterns

In Section 4.2 we described as part of hypothetical actions the mechanism of detecting deadly patterns; i.e. dynamically storing patterns in the danger memory $\mathcal {D}$ and avoiding them, without the need for human input. Figure 19 shows three characteristic examples, where such a technique is useful. In Fig. 19a the deadly pattern ${dp}=({Left}, {Right})$ is likely stored during the first step of ‘crossing the bridge’ and then checked all along the way. Similarly, in Fig. 19b once the deadly pattern ${dp}=({Left}, {Down})$ is stored in $\mathcal {D}$, it is continuously automatically detected throughout the path to the goal state. This means that not only are such patterns always avoided, but also the human is not bothered answering a preference query about this combination (although they do need to provide information about the other cells, as in HAs). For 1000 trials, using the conservative setting, both of these environments gave trends of training deaths and number of queries for Parenting, JPAL and JPAL-HA similar to the original configuration (Fig. 1). The only difference was now that in JPAL-HA $P_{{add}}^{({DP})}$ and $J_{a_{{new}}}^{({DP})}$ were comparable or even exceeded $P_{{add}}^{({BTFQ})}$ and $J_{a_{{new}}}^{({BTFQ})}$, respectively. Similar observations were made in Fig. 19c where the mechanism is even more useful, as once ${dp}=({Left}, {Right}, {Down})$ is detected, then only one $J_{a_{{new}}}^{({DP})}$ query about the fourth safe action is required when a pattern state is met, in order to map out the whole dangerous area. Thus, we exploit this technique to both improve safety and reduce the queries issued.

### D.2 Training in more Island Navigation maps

In this section we train on three more maps of the Island Navigation environment (Fig. 20) and investigate if the results agree with the conclusions from Sections 5.3, 5.4, 5.6, 5.8 and 5.9.

For the maps of Fig. 20a and b, the results are shown in Table 8 for the conservative setting ($p_{{BTFQ}}=0.95$, $p_{{REC}}=0.8$, $p_{{ATFQ}}=0.8$). We observe that trends remain mostly consistent with each other and consistent with the analysis in the original map (Fig. 1). For example, the training deaths decrease with the inclusion of justifications in JPAL and further decrease with the inclusion of hypothetical actions in JPAL-HA. The overall steps, feedback time and training time until optimality increase in JPAL, but then drop in JPAL-HA. The results from the map of Fig. 20a seem very consistent with the ones of the original map, but in Fig. 20b the positive impact of hypothetical actions is even stronger. That is, we see that for Fig. 20b the overall steps and preferences of JPAL-HA are even less than Parenting, and the decrease in feedback time and training time from JPAL is much more notable. The above has a reasonable explanation. All maps have the same dimension (7*×*9); Figure 1 has 11 cells not neighbouring with a water cell, Figure 20a has 10 cells (almost the same), but Fig. 20b has only one. Because JPAL-HA knows how to handle situations close to water more efficiently than Parenting and JPAL, its effect will be stronger.

**Table 8:** Average number of training deaths, preferences (*P*), justifications over preferences ($J_{P}$), justifications over hypothetical actions ($J_{a_{{new}}}$), human-feedback time, training time and overall steps for training in the configurations of Fig. 20a and b until the optimal policy is found; mean and standard deviation are reported over 200 trials. All trials used the conservative setting

	Configuration of Fig. 20a			Configuration of Fig. 20b		
	Parenting	JPAL	JPAL-HA	Parenting	JPAL	JPAL-HA
Training deaths	*0.10 ± 0.34*	*0.04 ± 0.21*	*0.03 ± 0.17*	*0.24 ± 0.46*	*0.14 ± 0.41*	*0.07 ± 0.27*
*P*	*40.06 ± 6.38*	*50.15 ± 10.26*	*46.29 ± 8.20*	*52.20 ± 6.12*	*55.10 ± 7.85*	*46.27 ± 6.03*
$J_{P}$	−	*50.15 ± 10.26*	*40.70 ± 7.85*	−	*55.10 ± 7.85*	*36.62 ± 5.60*
$J_{a_{{new}}}$	−	−	*9.65 ± 2.51*	−	−	*15.56 ± 2.85*
Feedback time (s)	*153.43 ± 24.45*	*249.75 ± 51.07*	*247.74 ± 44.54*	*199.93 ± 23.45*	*274.42 ± 39.10*	*257.43 ± 34.55*
Training time (s)	*258.41 ± 54.10*	*385.79 ± 90.18*	*366.07 ± 75.72*	*343.38 ± 60.24*	*433.32 ± 84.88*	*370.25 ± 62.99*
Overall steps	*52.49 ± 16.12*	*68.02 ± 20.59*	*59.16 ± 16.94*	*71.72 ± 20.07*	*79.45 ± 24.60*	*56.41 ± 16.49*

**Table 9:** Average number of training deaths, preferences (*P*), justifications over preferences ($J_{P}$), justifications over hypothetical actions ($J_{a_{{new}}}$), human-feedback time, training time and overall steps for training in the configuration of Fig. 20c until the optimal policy is found; mean and standard deviation are reported over 200 trials

	JPAL ($p_{{BTFQ}}=0.95$)	JPAL-HA ($p_{{BTFQ}}=0.95$)	JPAL-HA ($p_{{BTFQ}}=0.99$)
Training deaths	*0.07 ± 0.27*	*0.03 ± 0.17*	*0.00 ± 0.00*
*P*	*54.51 ± 16.57*	*47.63 ± 14.60*	*49.05 ± 14.87*
$J_{P}$	*54.51 ± 16.57*	*38.87 ± 12.49*	*40.02 ± 12.58*
$J_{a_{{new}}}$	−	*14.48 ± 5.38*	*14.83 ± 5.69*
Feedback time (s)	*271.46 ± 82.50*	*262.61 ± 80.53*	*270.24 ± 81.69*
Training time (s)	*409.22 ± 136.48*	*379.16 ± 129.59*	*390.70 ± 127.62*
Overall steps	*68.88 ± 28.05*	*58.27 ± 26.19*	*60.23 ± 24.59*

In the map of Fig. 20c we increased the dimension to 10*×*10, and placed a couple of water cells. We found that Parenting significantly struggled, yielding at least one death in 1/3 of the trials, and many deaths within some trials without being able to complete training within a predefined number of 15 episodes in some of them. So we do not compare the metrics directly with JPAL and JPAL-HA which had the expected behaviour. As we can see in the first two columns of Table 9, in the conservative setting ($p_{{BTFQ}}=0.95$) JPAL-HA had the usual improvement compared to JPAL in all metrics. Finally, to confirm that additional queries can decrease the training deaths even further, we ran JPAL-HA with $p_{{BTFQ}}=0.99$. As we can see in the last column, training deaths reached zero, which however required a small increase in the rest of the metrics representing human input and time. This is consistent with our findings regarding the trade-off between human burden and safety, i.e. that more queries will typically lead to better safety. We note, however, that only $p_{{BTFQ}}=1$ guarantees no deaths.^34^ Also, as we explained in the main text, Appendix A proposes a method based on confidence in a state or region, that aims to untangle the choice of $p_{{BTFQ}}$ from the nuances of the environment.

### D.3 Testing JPAL-HA in more environments

We also wanted to test JPAL-HA in some more gridworld environments (Fig. 21) from [15]. Even though the main problem of safe RL is safe exploration which was modelled by the Island Navigation environment (Fig. 1), there are more problems regarding the *agent alignment* problem (aligning the agent’s behaviour with the human intentions) [14] that sometimes need to be considered. Since these problems mainly derive from a misspecified environmental reward function and JPAL-HA does not use such a reward, it was unlikely to face issues with these problems. However, we tried two of them.

Figure 21a illustrates the *unsafe interruptibility* problem modelled by the Off-switch environment, where a traditional RL agent learning to go towards the goal is interrupted by 50% chance at the orange cell and receives a -1 reward until the end of an episode that it is interrupted. That would incentivise it to go to the blue cell and press the button, which would disable the interruption. However, in the real world an interruption like that might be useful for the reasons of repair, upgrade, recharge the battery, etc. JPAL-HA which is driven by a human in only a few episodes learns to always avoid the blue cell and go immediately towards the goal, for the reason that there is no reward in an episode that an interruption needs to be done.

Finally, the Absent Supervisor environment in Fig. 21b is an abstraction of the *absent supervisor* problem. Specifically, in the presence of a supervisor, the RL agent would go right taking the long route to the goal not to receive a punishment of -30 at the yellow cell. However, knowing that the supervisor is absent, it would take the short one knowing that there is no punishment. The supervisor is present with probability 50%. With JPAL-HA the agent does not get tempted to take the risky short route as the human teaches it to take the safe long one. As we observed at deployment, after a few preference queries it never took the short way of punishment even when the supervisor was absent.

## Appendix E Current and future impact

In an outreach activity at the Southampton Science and Engineering Festival 2022, 60 participants, including both children and adults, successfully trained the Thymio robot [28] using the JPAL-HA model in a slightly smaller gridworld than that of Fig. 1. Similarly did nine high-school students aged between 16 and 19 during an educational trip at the University of Southampton. Participants could interact both via clicking on the laptop’s interface or via natural language through a headset microphone. We observed an easy grasp of the training procedure from people of all ages and background knowledge and contributed to the public awareness of human-in-the-loop AI, human-agent collaboration, AI safety and other related notions.

RL is a very strong tool, but careless use of it can have unpredictable results. Accidents that have involved autonomous systems in the past have decreased the public’s trust in them. All applications involving a learning agent for decision-making should take into account safety. The ideas and frameworks proposed in this paper, using a human in a standardised way to improve safety, point towards that direction of increasing the public’s trust. Moreover, even though robots will most likely replace a lot of human jobs in the future, the human-agent interface we are proposing is directed at employing people to train agents efficiently. Apparently, people would be able to train intelligent agents with the only requirement to understand the environment reasonably well and be able to answer preference and justification queries.

## Footnotes

1 Even though we simulate experiments to draw conclusions, we assume they should run in the real world, with a real human and robot in order to avoid the reality gap as done in Section 5.8.

2 Our method only uses this reward function for evaluation.

3 We change the terminology and make some technical improvements to Parenting [11], described in Sections 3, 4.3 and 5.1; these optimisations are, however, irrelevant to the core contributions of justifications and HAs. When referring to Parenting, we mean the optimised version.

4 $a^{*}$ is stored simply to avoid bothering the human with the same queries and also avoid multiple copies in $\mathcal {X}$. For an identical previously-asked query, the agent will be able to retrieve the answer from $\mathcal {X}$. This mechanism was not mentioned to be implemented in [11].

5 This mechanism, although inspired by [11], has been significantly modified and is described in detail in Section 4.3.

6 The idea behind using the term *justifications* is that the user provides a rationale for the expressed preference. In this context we only consider warnings regarding one safety aspect (water); however, there may be more (e.g. avoid a nearby pedestrian), and not of the same severity (e.g. limited battery). Appendix B describes how the idea of justifications (presented in Section 4.1 only for warnings regarding one safety factor) can be generalised to an arbitrary number of justifications.

7 There is a difference in Lines 2 and 3 with Algorithm 1, where $J_{P}$ is not provided as human input and used to shape the preference label *μ*.

8 Appendix B demonstrates how $\mu \in \{0, 0.25, 0.5, 0.75, 1\}$ can be understood as a specific realisation of a more general framework that uses *multiple justifications* with a different *weight for each justification*. For the case of one safety justification and a default one, as presented here, we have $\mu \in \{1-w_s, 1-w_{def}, 0.5, w_{def}, w_{s}\}$, where $w_{s}$ is the safety justification weight for water and $w_{def}$ is the default justification weight for moving faster to the goal cell. Thus, we effectively select the sensible choice of $w_{s}=1$ and $w_{def}=0.75$, giving more importance to safety.

9 We will return to the reason it is left blank when the loop is finished.

10 For continuous spaces, the approach could involve discretising the space into manageable regions or applying density estimation techniques, such as kernel density estimation [19], to assess the distribution of safe and catastrophic actions, ensuring sufficient exploration coverage before stopping.

11 We immediately witnessed a significant improvement to the results of Parenting after optimising it, which was probably because Parenting from [11] explored carelessly (as described in Section 4.3) and additionally trained the agent only for 1 epoch per step. The results we report are from the optimised Parenting.

12 For example, in such safe RL methods sample efficiency is usually measured by environment interactions, whereas in the human-in-the-loop methods we also need to consider the aspect of human burden, measured through the number and type of queries. Thus, even if we could observe lower overall interactions with the human-based methods, we would not be able to claim superiority under the same assumptions.

13 The *discount \ factor* was chosen after experimentation, the *learning \ rate* was 1 as this is the optimal value for deterministic environments and the starting *ε* was set to a small value, in order to keep deaths as low as possible.

14 Parenting and JPAL have more subtle differences to JPAL-HA at a smaller scale than the one illustrated in Fig. 4 (their curves would overlap). These differences are presented in detail in Section 5.3. The magenta curve of HIRL [22] will also be explained in Section 5.7.

15 We report the mean and 1 standard deviation values $\mu \pm \sigma$ for this and the next experiments.

16 Since we worked with a small environment, this is what we did for convenience to assess that the optimal policy was found and a trial was over; we deployed the agent with the greedy policy and checked if it followed the shortest path to the goal.

17 Apparently, there is a rare case (encountered for very few seeds) of a catastrophe after optimality, which may occur when the two sampled actions do not include the greedy one that leads to the goal (unlikely since the policy has already been shaped), so the agent starts going through completely new routes. A candidate workaround to this would be one of the two sampled actions to always be the greedy one after some advanced point in time.

18 Unlike other variables that the standard deviation is lower than the mean, regarding the number of training deaths the standard deviation is higher because deaths are rare having been averaged over 1000 trials.

19 Until Section 5.6 we do not consider ATFQs which as it will be described in Section 5.5 are not urgent to be issued and also the differences in their number amongst methods are marginal.

20 We will return to this argument in Section 5.6.

21 Although it made a small difference in the overall training steps and queries, this is also the reason we made the temporary memory *R* a stack (instead of picking randomly a recording), so that the agent could record and issue to the human an escaping ATFQ as fast as possible.

22 Although it is not specified explicitly in [22] whether the human replaces the dangerous action with the optimal or just a safe one, for our experiments we replaced it with the optimal.

23 In Section 5.9 we also compare them more directly with reference the actual time.

24 As explained in Section 2, in this work we also did not model the human error (assuming users can understand the environment and give correct answers); thus, we used a simple autocorrection mechanism to prompt users to correct their answer in case of an oversight, while the clock continued running. Modelling human error is discussed in Section 6.2.

25 All experimental protocols were approved by University of Southampton Ethics and Research Governance Online (ERGO), the methods were carried out in accordance with the relevant guidelines and regulations, and informed consent was obtained from all participants.

26 Since there was only one labeller, the HIRL average response times should be interpreted as approximate, and possibly slightly favourable for HIRL, since the labeller was well-familiar with the GUI. However, they are still useful for placing HIRL within the broader comparison with the other methods.

27 The region at the beginning of the curves where the training return sometimes starts high and then drops until the point that it starts going up again (most prominent in HIRL) is because a mean training return may be computed over not all trials, but only by the ones that at least one episode had been completed by that time budget. In those trials with an early-completed episode, the agent asks queries that lead it quicker towards the goal, so they yield higher return in less time. As more trials, with an episode that took more time to complete, enter and contribute to the average, the latter drops before it starts increasing again until convergence.

28 An analogue is the example of a driving instructor. Parenting is like a loose driving instructor who hastily tries to teach a student how to drive within a few hours, while JPAL or JPAL-HA is like a careful instructor who devotes more input and time to learning. If teaching stopped at the same time before the students had become experts in driving and asked them to drive by themselves, the student of the loose instructor may indeed know how to drive better and more safely. However, the loose teacher would jeopardise more accidents during the learning process. In contrast, with the careful teacher, not only the student would finally drive well and safely, but also there would not be dangers during learning.

29 For example, in a questionnaire that people mark a service from 1 to 10, it may be confusing what 7, 8 or 9 means; instead a much easier question allowing a concrete answer is ‘do you prefer service A or service B?’.

30 Earlier GPTs, such as GPT-3 [58] did not use human preferences, so although powerful, they lacked alignment with human objectives. In the case of LLMs, ‘alignment’ refers to outputs from the chatbot that are truthful, untoxic and helpful — components directly correlated to safety that could be only achieved with the human input during training.

31 Part of this direction, specifically utilising justifications within a world model and deploying with MPC, has been explored in subsequent work through the DROPJ framework, further details of which can be found in [87, 88].
